# Bioinformatics-Guided Identification and Quantification of Biomarkers of *Crotalus atrox* Envenoming and Its Neutralization by Antivenom

**DOI:** 10.1016/j.mcpro.2025.100956

**Published:** 2025-03-25

**Authors:** Auwal A. Bala, Anas Bedraoui, Salim El Mejjad, Nicholas K. Willard, Joseph D. Hatcher, Anton Iliuk, Joanne E. Curran, Elda E. Sanchez, Montamas Suntravat, Emelyn Salazar, Rachid El Fatimy, Tariq Daouda, Jacob A. Galan

**Affiliations:** 1 Department of Human Genetics, School of Medicine, University of Texas Rio Grande Valley, Brownsville, Texas, USA; 2 Faculty of Medical Sciences, UM6P Hospitals, Mohammed VI Polytechnic University, Benguerir, Morocco; 3 National Natural Toxins Research Center (NNTRC), Texas A&M University-Kingsville, Kingsville, Texas, USA; 4 Department of Chemistry, Texas A&M University-Kingsville, Kingsville, Texas, USA; 5 Tymora Analytical Operations, West Lafayette, Indiana, USA

**Keywords:** snakebite, antivenom, systems biology, proteomics, extracellular vesicles

## Abstract

Quantitative mass spectrometry-based proteomics of extracellular vesicles (EVs) provides systems-level exploration for the analysis of snakebite envenoming (SBE) as the venom progresses, causing injuries such as hemorrhage, trauma, and death. Predicting EV biomarkers has become an essential aspect of this process, offering an avenue to explore the specific pathophysiological changes that occur after envenoming. As new omics approaches emerge to advance our understanding of SBE, further bioinformatics analyses are warranted to incorporate the use of antivenom or other therapeutics to observe their global impact on various biological processes. Herein, we used an *in vivo* BALB/c mouse model and proteomics approach to analyze the physiological impacts of SBE and antivenom neutralization in intact animals; this was followed by bioinformatics methods to predict potential EV biomarkers. Groups of mice (n = 5) were intramuscularly injected with Saline or *Crotalus atrox* venom. After 30 min, the mice received saline or antivenom (Antivipmyn) by intravenous injection. After 24 h, blood was collected to extract the plasma to analyze the EV content and determine the exposome of *C. atrox* venom as well as the neutralizing capabilities of the antivenom. The predicted biomarkers consistently and significantly sensitive to antivenom treatment are Slc25a4, Rps8, Akr1c6, Naa10, Sult1d1, Hadha, Mbl2, Zc3hav, Tgfb1, Prxl2a, Coro1c, Tnni1, Ryr3, C8b, Mycbp, and Cfhr4. These biomarkers pointed toward specific physiological alterations, causing significant metabolic changes in mitochondrial homeostasis, lipid metabolism, immunity, and cytolysis, indicating hallmarks of traumatic injury. Here, we present a more comprehensive view of murine plasma EV proteome and further identify significant changes in abundance for potential biomarkers associated with antivenom treatment. The predicted biomarkers have the potential to enhance current diagnostic tools for snakebite management, thereby contributing significantly to the evolution of treatment strategies in the diagnosis and prognosis of SBE.

## Highlights

• Mass spectrometry-based proteomics of mice plasma extracellular vesicles.
• Murine plasma exosomes contain proteins sensitive to envenoming and antivenom.
• Protein abundance changes in plasma extracellular vesicles post venom exposure.
• Snake venom causes changes in lipid metabolism, hemostasis, and immune response.
• Potential diagnostic and prognostic protein biomarkers of snakebite envenoming.

## Graphical Abstract

**Figure:**
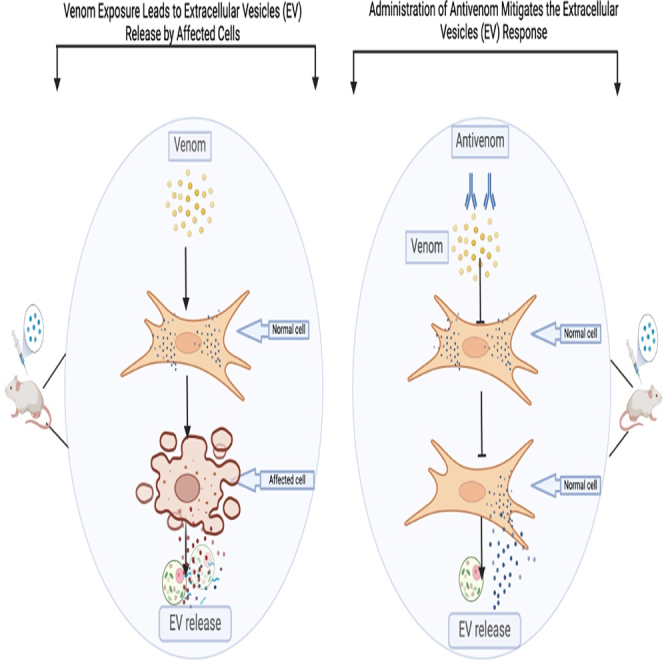

## In Brief

This study focus on mice plasma extracellular vesicles (EV) released after mice exposure to snake venom. We exposed mice to *Crotalus atrox* whole venom and antivenom intravenously and then checked the plasma EV response through their protein cargo. Here is an overview of murine plasma EV proteome with significant changes in protein abundance for potential biomarkers associated with venom and antivenom exposure. These have the potential to enhance current diagnostic tools for snakebite management, thereby contributing to the diagnosis and prognosis of snakebite envenoming.

Snake venom is composed of various complex protein-based toxins that make up 12 major protein families (1). Snake venom toxins can disturb enzyme and receptor/ion channel activities to impact tissue integrity as well as the nervous and hemostatic systems with a high degree of target specificity (2, 3, 4). Each venom composition is quite specific to the snake family, and exploring the regulation and mutation of snake venom toxins may reveal adaptive variations in phenotypes and aid in the development of therapeutic substances (2, 5). The World Health Organization has declared snakebite envenoming (SBE) a globally neglected tropical disease resulting from about 5.4 million cases every year. Roughly 2.7 million of these cases are correlated to envenomings, leading to an average of 138,000 deaths with 400,000 disabilities annually (6, 7, 8, 9, 10, 11). Thus, controlling and preventing snakebites is a major issue that warrants developing strategies to ensure safe and effective antivenoms (6, 12, 13, 14).

The United States records about 10,000 emergency snake envenoming cases annually, and approximately 4500 of these are caused by Crotalinae snakes (8). Envenoming involves endogenous processes that contribute to pathophysiology by activating downstream cascading events such as inflammation, pain, and hemodynamic mediation (15, 16). Numerous intracellular/extracellular damage-associated molecular patterns (DAMPs or alarmins) are also created, impacting the innate immune system to generate mediators with multiple actions (17, 18). Venom-associated molecular patterns also directly activate the innate immune system (19, 20) and reactive oxygen species found in affected tissues (21). *Crotalus atrox,* known as the Western diamondback rattlesnake, is responsible for most envenomings in Northern Mexico and the Southwestern United States. *C. atrox* is listed as a species of high medical importance (22), and its venom toxins have hemotoxic, myotoxic, cytotoxic, and hemorrhagic activities (23, 24, 25). The snake venom metalloproteinases (SVMP) are the most abundant protein family, making up about 50% of the total venom composition (26). Antivenom effectiveness depends on the immunogenicity of proteins used in the immunization process and the ability of the antivenom to reverse venom-induced pathophysiology (27, 28). Limitations of antivenom treatment include the requirement of trained medical personnel to administer treatment, the time delay in transport from the site of inoculation to the health care facility (29, 30, 31, 32), local acute damage when left untreated (33, 34, 35), the cost of antivenom manufacturing, availability, accessibility in rural lands, lack of reactivity across species, venom specificity from immunological variation (36, 37, 38), and cold storage requirements (7). Early administration of antivenom can assist in preventing severe pathologies of SBE. However, there are limitations in understanding which specific markers or biological pathways are perturbed and which can be mitigated or restored by antivenom treatment.

Systems biology studies provide insight into the properties and behaviors of complex and dynamic biological information on a systemic level to understand the signal transduction for regulating and coordinating networks in a pathophysiological response (39). Bioinformatics has played an important role in discovering and predicting biomarkers, significantly advancing our understanding of snakebite impacts. Combining these concepts yields an approach that provides highly specific biomarkers and drug targets, predicting and identifying key genes/proteins related to signaling pathways, suggesting that the signaling networks give specific systemic signatures in different diseases and/or treatments (40). This kind of analysis views the global proteome as well as enriched proteins/peptides in biological systems and enables the identification of upregulated/downregulated biological processes (41). The worldwide study of the effects of SBE on the body necessitates applying numerical multiomics methods to understand snake venom when it gets into the bloodstream. These methods shed light on the venom's protein composition, but more investigation is needed to examine the venom-exposome and pinpoint specific markers of snake venom and antivenom treatment. Predictive biomarkers have emerged as essential components in this exploration. They allow us to comprehend specific pathophysiological changes that occur post envenoming and post treatment, providing critical insights into the venom’s and antivenom's impact on the host's system.

The recent finding of extracellular vesicles (EVs) and their vital roles in cellular processes has highlighted their potential as a novel source for identifying biomarkers and diagnosing diseases. EVs are important for cell communication, apoptosis, immune responses, and various other biological processes (42) and have been isolated from biological fluids such as blood, urine, saliva, and breast milk (43). They have recently been gaining some attention due to their recent offerings of new promising biomarkers for detecting various diseases such as cancer, neurodegenerative, and cardiovascular disease (44, 45, 46, 47). Recently, we have studied the EVs from *C. atrox* and *Crotalus oreganus helleri* snakes, and identified new signaling, adaptor, transmembrane, and vesicle proteins using an EVtrap enrichment method (48, 49, 50). The additional analysis of mouse plasma-derived EVs by quantitative mass spectrometry revealed insights into the murine plasma EV response post envenomation and identified potential biomarkers involved with the altered metabolic pathways from envenoming. The information may be analyzed by capturing the EVs released by cells into the plasma and reading their contents. Through the use of mouse plasma-derived EVs, the pathophysiological response to SBE and the efficiency of treatment methods may be quantified (48). This integrated approach opens avenues for evaluating potential antivenoms and other therapeutics. It provides a global perspective on the impact of these interventions on various biological processes disrupted by the venom, improving the precision and efficacy of treatments.

The results herein provide a systems biology approach to studying the pathophysiological aspects and the exposome of toxicity in SBE by analyzing the contents of EVs extracted from blood plasma using mass spectrometry and bioinformatics. We used an *in vivo* BALB/c mouse model, EV isolation, and proteomics approach to analyze the physiological impacts of *C. atrox* envenoming and antivenom (Antivipmyn) neutralization, then predicting biomarkers using different bioinformatic methods. We found significant changes in protein abundance which might indicate biological/metabolic changes by envenoming that were neutralized by antivenom, demonstrating a novel approach to monitoring snake venom therapeutics in acute response conditions. Our results suggest the capability for a unique and novel model and method to study snake venom and antivenom interactions and potential hypersensitivity reactions *in vivo*. Incorporating these biomarkers into “omics” studies, including genomics, transcriptomics, proteomics, and metabolomics, is ushering in a new era of snakebite research.

## Experimental Design and Statistical Rationale

### Experimental Design

Four groups of BALB/c mice (M & F, 18–20 g) were intramuscularly injected with a primary dose of 200 μl saline or *C. atrox* venom (lx LD_50_, *102 μg/mouse*). After 30 min, the mice received a secondary i.v. injection of 200 μl saline or Antivipmyn, F(ab’)_2_ polyvalent antivenom (prepared by manufacturer instructions). One mouse was ethically excluded from the treatment group due to difficulty detecting a vein for intravenous injection, and the statistical value for the missing mouse was imputed using Perseus software; the software imputes missing values by drawing random numbers from a normal distribution of 1.8 standard deviation down shift and with a width of 0.3 of each sample. Thus, the grouping was as follows: saline/saline group, n = 5 (3M, 2F), saline/venom group, n = 5 (3M, 2F), saline/antivenom group, n = 5 (3M, 2F), and venom/antivenom group, n = 4 (2M, 2F). After 24 h of the secondary injection, mice were sacrificed by cervical dislocation, and blood was extracted *via* cardiac puncture. Approximately 0.3 ml of mouse blood was placed in a 1.5 ml Eppendorf microfuge tube containing EDTA and centrifuged at 1000*g* for 10 min at 4 °C to collect the separated plasma. Plasma was collected and stored at −80 °C. Mouse plasma EVs were isolated using EVtrap as previously described (49, 50). Magnetic EVtrap beads were added to the plasma samples at a ratio of 1:100 v/v, and the mixtures were incubated for 1 h with shaking. The supernatant was removed with a magnetic rack and washed with PBS. EVs were eluted with 100 mM of fresh triethylamine in two 10-min incubations. This was followed by drying with a vacuum centrifuge, and proteins from the dried EVs were extracted using the standard phase-transfer surfactant aided procedure. Briefly, EVs were solubilized in the lysis solution containing 12 mM sodium deoxycholate, 12 mM sodium lauroyl sarcosinate, 10 mM tris(2-carboxyethyl) phosphine hydrochloride, 40 mM CAA, and phosphatase inhibitor cocktail in 50 mM Tris–HCl, pH 8.5 by incubating 10 min at 95 °C. This step also denatured, reduced, and alkylated the proteins. The samples were diluted five-fold with 50 mM triethylammonium bicarbonate and digested with trypsin at 1:50 (w/w) enzyme-to-protein ratio for overnight digestion at 37 °C. To the digested peptides, ethyl acetate solution was added at a 1:1 ratio, and the samples were acidified with TFA to a final concentration of 1% TFA. The mixture was vortexed for 2 min and then centrifuged at 20,000*g* for 2 min to obtain aqueous and organic phases. The organic phase (top layer) was removed, and the aqueous phase was collected, dried down in a vacuum centrifuge, and desalted using 100-mg Sep-Pak C18 columns (Waters) according to the manufacturer’s instructions.

### Statistical Rationale

To ascertain statistical significance, we implemented multiple statistical tests strategy, bolstering the robustness of our estimates and controlling the rate of false discoveries. An initial Student’s *t* test was conducted to compare the mean protein expression levels across various conditions. To accommodate the nonnormal distribution of protein expression data and potential outliers, we complemented this with permutation shuffle testing (1000 simulations). To control multiple tests, the Benjamini-Hochberg procedure was used. Additionally, we established a threshold Univariate Logistic regression accuracy score of >80%. By intersecting the significant proteins from these three tests, we ensured the selected proteins exhibited significant differences in mean abundance (*t* test), maintained these differences under a nonparametric statistical framework (permutation test), and provided valuable predictive information about the condition (logistic regression). To identify venom biomarker identification, Venom condition was tested against saline and antivenom conditions. We prioritized the top proteins—determined by fold-change relative to the saline mean—within the overlapping set of significant proteins identified through our multitest approach. For identifying biomarkers sensitive to antivenom, we first identified proteins with the highest significant fold-change between the venom and saline conditions. From this subset, we further selected those showing the most contrasting significant fold-change between the treatment and venom conditions. All biomarker prediction analyses were conducted using the Python programming language. The raw dataset underwent a series of preprocessing steps. Initially, proteins absent in all samples were deemed noninformative and eliminated. Subsequently, for the remaining proteins, any missing expression levels in particular samples were imputed with the median expression level of that protein across all other samples. After imputing missing values, we applied a log2 transformation and Z-standardization to the protein abundances to ensure a comparable scale. Finally, only the top 95% of proteins based on variance across samples were retained.

## Experimental Procedures

### Ethical Approval

Ethical approval was obtained from the Texas A&M University-Kingsville Institute of Animal Care and Use Committee (09-11-2018, #2018-11-09-A3).

### Venom Collection and Antivenom

Lyophilized venom of *C. atrox* was obtained from the Serpentarium of National Natural Toxins Research Center, Texas A&M University-Kingsville, Kingsville, TX. It was concentrations designated as *C. atrox* vial 946 (AVID# 065-091-774). Antivipmyn was obtained from Instituto Bioclon, Mexico, and the protein concentration was determined by extinction coefficient using a spectrophotometer; an absorbance at 280 nm of 1.4 equals 1.0 mg of immunoglobulin G.

### Proteomic Analysis (Liquid Chromatography Mass Spectrometry)

One microgram of each dried peptide sample was dissolved in 10.5 μl of 0.05% TFA with 3% (vol/vol) acetonitrile. In total, 10 μl of each sample was injected into an Ultimate 3000 nano UHPLC system (Thermo Fisher Scientific). The peptides were captured on a 2 cm Acclaim PepMap trap column and separated on a heated 50 cm column packed with ReproSil Saphir 1.8 μm C18 beads (Dr Maisch GmbH, Ammerbuch, Germany). The mobile phase buffer was composed of 0.1% formic acid in ultrapure water (buffer A) with an eluting buffer of 0.1% formic acid in 80% (vol/vol) acetonitrile (buffer B) and ran with a linear 60 min gradient of 6 to 30% buffer B at a flow rate of 300 nl/min. The UHPLC was coupled online with a Q Exactive HF-X mass spectrometer (Thermo Fisher Scientific). The mass spectrometer (MS) was operated in the data-dependent mode, in which a full-scan MS (from m/z 375–1500 with the resolution of 60,000) was followed by MS/MS of the 15 most intense ions (30,000 resolution; normalized collision energy—28%; automatic gain control target—2E4: maximum injection time—200 ms; 60 s exclusion). The raw files were searched directly against the *Crotalus* or *Mus musculus* available in UniProt with no redundant entries, using Byonic (Protein Metrics) and SEQUEST search engines loaded into Proteome Discoverer 2.3 software (Thermo Fisher Scientific). The searches were combined database and were used for reverse decoy for false discovery rate (FDR) estimations. MS1 precursor mass tolerance was set at 10 ppm and MS2 tolerance was set at 20 ppm. Search criteria included a static carbamidomethylation of cysteines (+57.0214 Da) and variable modifications of oxidation (+15.9949 Da) on methionine residues and acetylation (+42.011 Da) at the N terminus of proteins. The search was performed with full trypsin/P digestion and allowed a maximum of two missed cleavages on the peptides analyzed from the sequence database. The FDRs of proteins and peptides were set at 0.01. All protein and peptide identifications were grouped, and any redundant entries were removed. Only unique peptides and unique master proteins were reported (48). Data were quantified with a Proteome Discoverer v2.3 using the label-free quantitation approach, and the intensity was used to calculate abundance in the quantification results as described in (48). Peptide intensities were extracted with an initial precursor mass tolerance of 10 ppm, a maximum retention time (RT) of isotope pattern multiplets of 0.2 min, a peptide-spectrum match confidence FDR of 0.01, an ANOVA hypothesis test, a maximum RT shift of 5 min, pairwise ratio-based ratio calculation, and 100 as the maximum allowed fold change. To compare proteins within different samples, fold change between protein groups, r, and ratios of total protein abundance sums were calculated. All ratios showing a 2-fold change (ratio ≥2.0 or ratio ≤0.5) were considered biologically significant. The distributions of peptides and proteins abundance were synthesized with Excel and transferred to Perseus software (Version 2.0.3.1) for analysis and visualization as described by (48). Briefly, in the “Main” box, the abundance ratios, the individual abundances of the venom, and the control of the snake venoms were inserted. In the “numerical” box, the #peptides, #PSMs, # unique peptides, MW [kDa], calculated pI, and coverage [%] were placed. In the “categorical” box, modifications and accessions were placed. In the “Text” box, the gene name and description were inserted. Enrichment analysis was performed with functional enrichment (FunRich), Database for Annotation, Visualization and Integrated Discovery (DAVID), and Search Tool for the Retrieval of Interacting Genes/Proteins (STRING) Analysis tools.

## Results

### Experimental Workflow

**Fig. 1:**
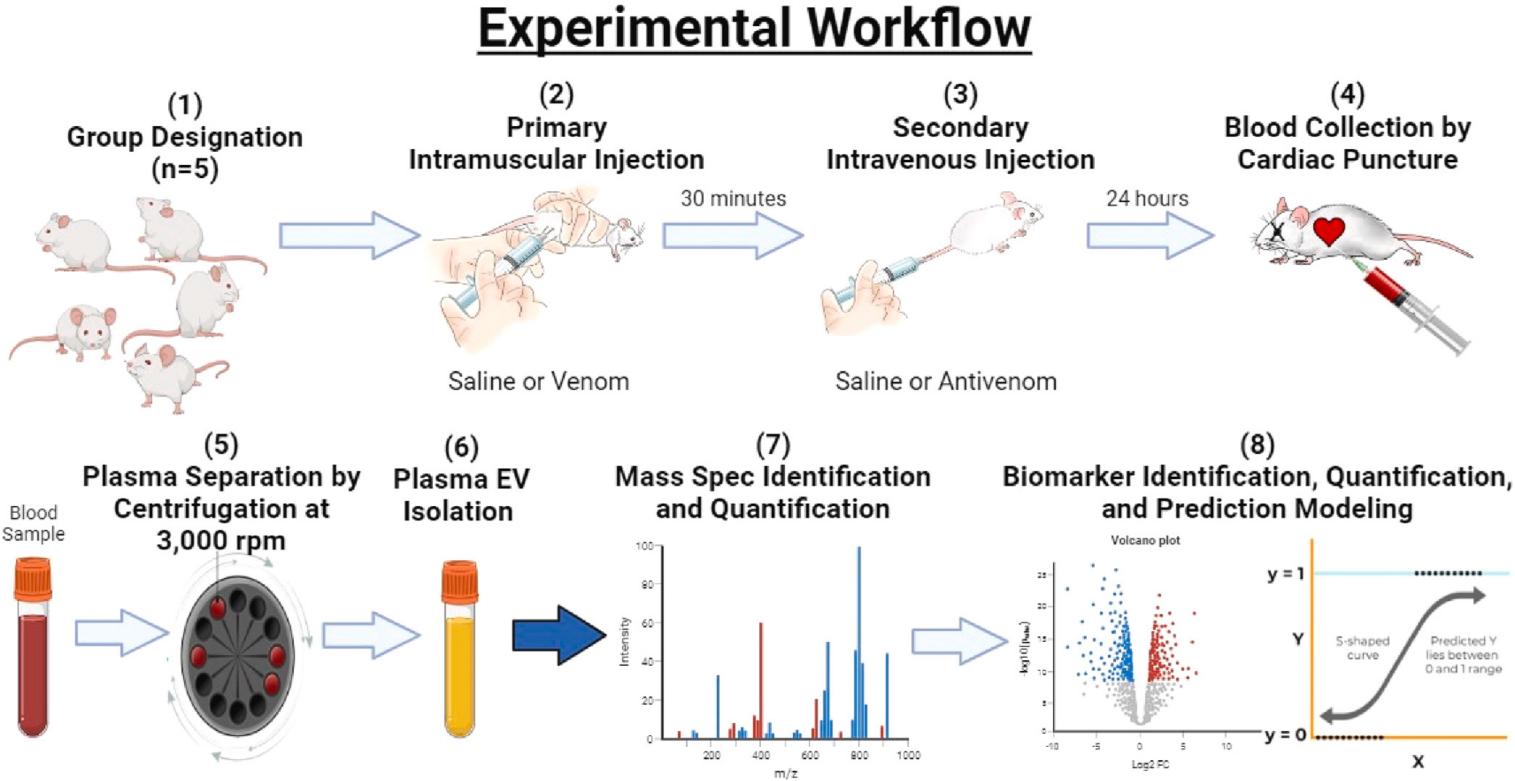
**Schematic representation of the procedure.** 1. design of the four different conditions with n = 5 for each group. 2. each group received a primary thigh i.m. injection of either saline or venom (1x LD_50_ i.v. = *102 μg/mouse*). 3. a secondary tail vein i.v. of either saline or antivenom (prepared by manufacturer instructions; *17,200 μg/mouse*) after 30 min of the primary i.m. injection. 4. blood was collected by cardiac puncture with syringes coated with 0.05 M EDTA after 24 h of the secondary i.v. injection. 5. samples were loaded into a centrifuge at 3000 rpm for 10 min at 4 °C. The plasma was collected separately and stored at −80 °C until the next step. 6. EV isolation with the EVTrap technique. 7. mass spectrometry analysis to identify the EV protein content. 8. various proteomic software utilized for data analysis. i.m., intramuscular; EV, extracellular vesicle.

Using a workflow similar to our previous work (48), we designed an experiment comparable to a rescue assay (n = 5 for each condition) to analyze EVs in the systemic circulation of BALB/c mice treated with *C. atrox* venom followed by antivenom (Fig. 1). The objective of the analysis was to quantify changes in protein abundance from mice EVs after intramuscular injection with *C. atrox* venom followed by intravenous (i.v) injection of antivenom, which was assessed after 24 h, compared to saline controls used for venom and antivenom, respectively.

### Overall Proteomic Identifications and Quantitative Analysis

**Fig. 2:**
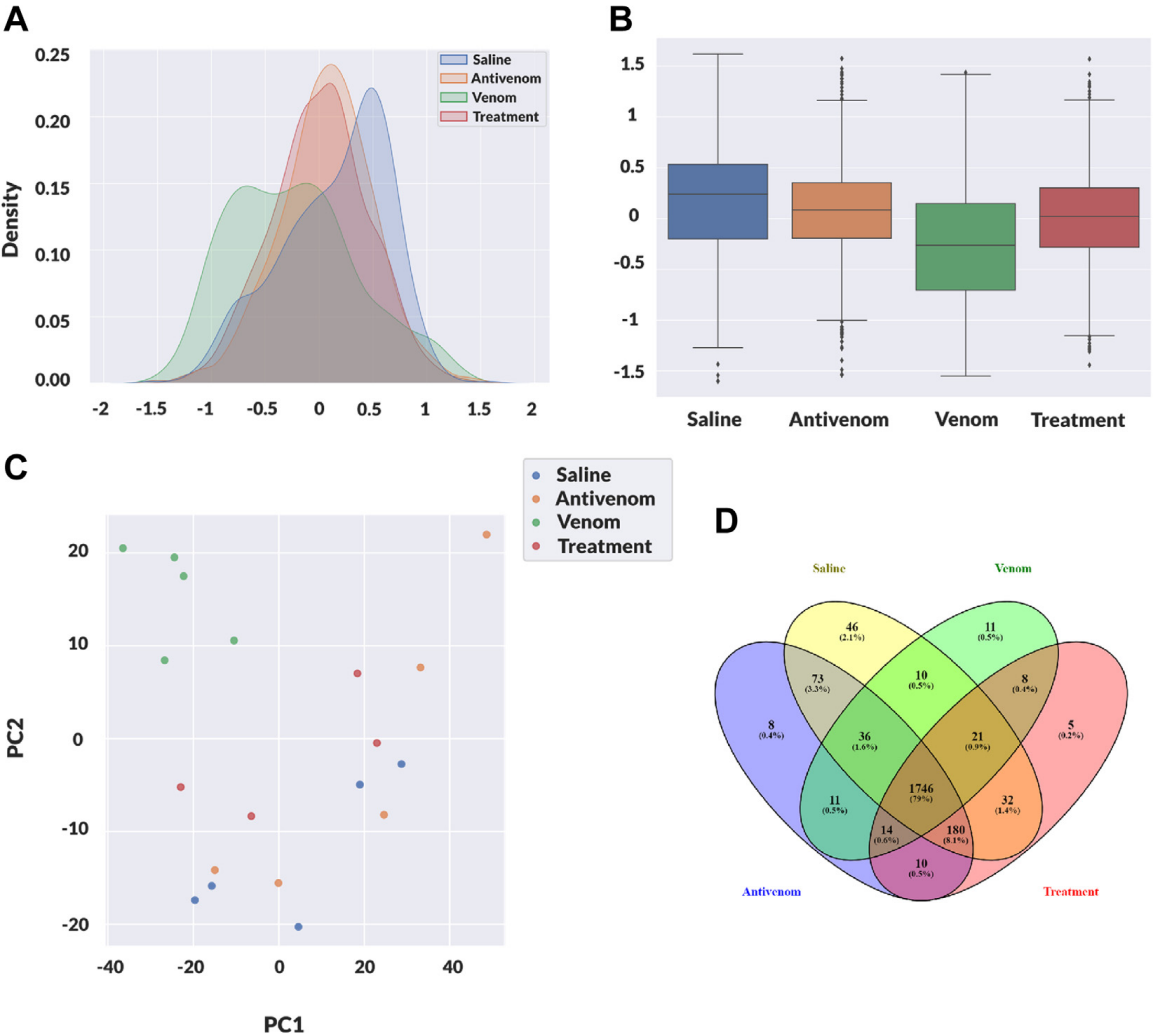
**Proteomic characterization of blood plasma EVs from saline, venom, treatment, and antivenom control.***A*, distribution of protein abundances for each of the four mouse conditions: saline, venom, treatment, and antivenom. Each density curve within the plot corresponds to one of these conditions, representing the mean of standardized protein abundances (in log2 scale) across samples for that condition. The venom condition showcases a unique distribution pattern. In contrast, the distribution of the treatment condition more closely mirrors those of the saline and antivenom conditions. *B*, box plot of protein abundance for each condition. Venom condition (*green*) has a distinct distribution from the other conditions. Treatment (*red*) shifts back the distribution closer to saline and antivenom conditions, in line with the restoration effect of the antivenom. *C*, PCA analysis of saline (*blue*), venom (*green*), antivenom (*orange*), and treatment (*red*) conditions. *D*, Venn diagram comparing the overlapping and unique proteins identified in the treatment (*red*), venom (*green*), antivenom (*blue*), and saline (*yellow*) conditions. EV, extracellular vesicle; PCA, principal component analysis.

Mass spectrometry-based proteomic analysis of the plasma EV samples identified and quantified 2642 unique murine proteins across all conditions without *Crotalus* specific proteins. The saline condition consisted of 20,239 peptides, 17,578 unique to 2144 proteins. The antivenom condition revealed 20,045 peptides, 17,457 unique peptides, and 2078 proteins. The venom condition revealed 19,482 peptides detected, and label-free quantification showed that 16,996 were unique to 1857 proteins. The treatment condition identified 19,880 peptides and quantified 17,373 unique peptides that correlated to 2016 proteins (supplemental Material S1). The distribution patterns of protein abundance levels for the 19 samples treated with venom, saline, and antivenom were found to be consistent and reliable (supplemental Material S2). Additionally, the correlation between pairs of samples was presented with the color intensity reflecting the correlation magnitude on the heatmap, and they all fall within a minimum of 0.75 to a maximum of 1.00, as indicated by the color scale (supplemental Material S3). The proteomic identification, enrichment, and quantitative changes observed in the samples of the "Venom" condition (venom alone) and the "Treatment" condition (venom followed by antivenom) showed notable differences, suggesting not only perturbations of the venom but also the effectiveness of the antivenom treatment mitigation, restoring the venom-induced changes similar to baseline (Saline condition). Under the treatment condition, the samples resembled the baseline seen in the mice of the saline condition, which served as the control group (Fig. 2*A*). Interestingly, the antivenom condition (antivenom alone) overlaps with the treatment condition, and they are closely similar to the saline condition but appear entirely different from the venom-alone condition (Fig. 2*A*). This suggests that the antivenom modulates the effects of the snake venom, returning the mice to a similar baseline (saline conditions). Similarly, the protein abundance of the venom condition showed a different distribution pattern compared with the treatment condition, which shifted back toward the saline condition (Fig. 2*B*). Although we observed 748 "single hit" protein IDs, out of 2642, 90 percent of the proteins that exhibited significant shifts in protein abundance levels had more than two peptide hits for identification. The principal component analysis (Fig. 2*C*) visualizes the proteomics data from the saline, venom, treatment, and antivenom conditions, showing distinct profiles between venom and other conditions. The Venn diagram in Figure 2*D* shows the number and percentage of proteins detected for each condition, visually representing unique and shared identifications.

### Significant Proteins Sensitive to Venom and Antivenom Treatment

**Fig. 3:**
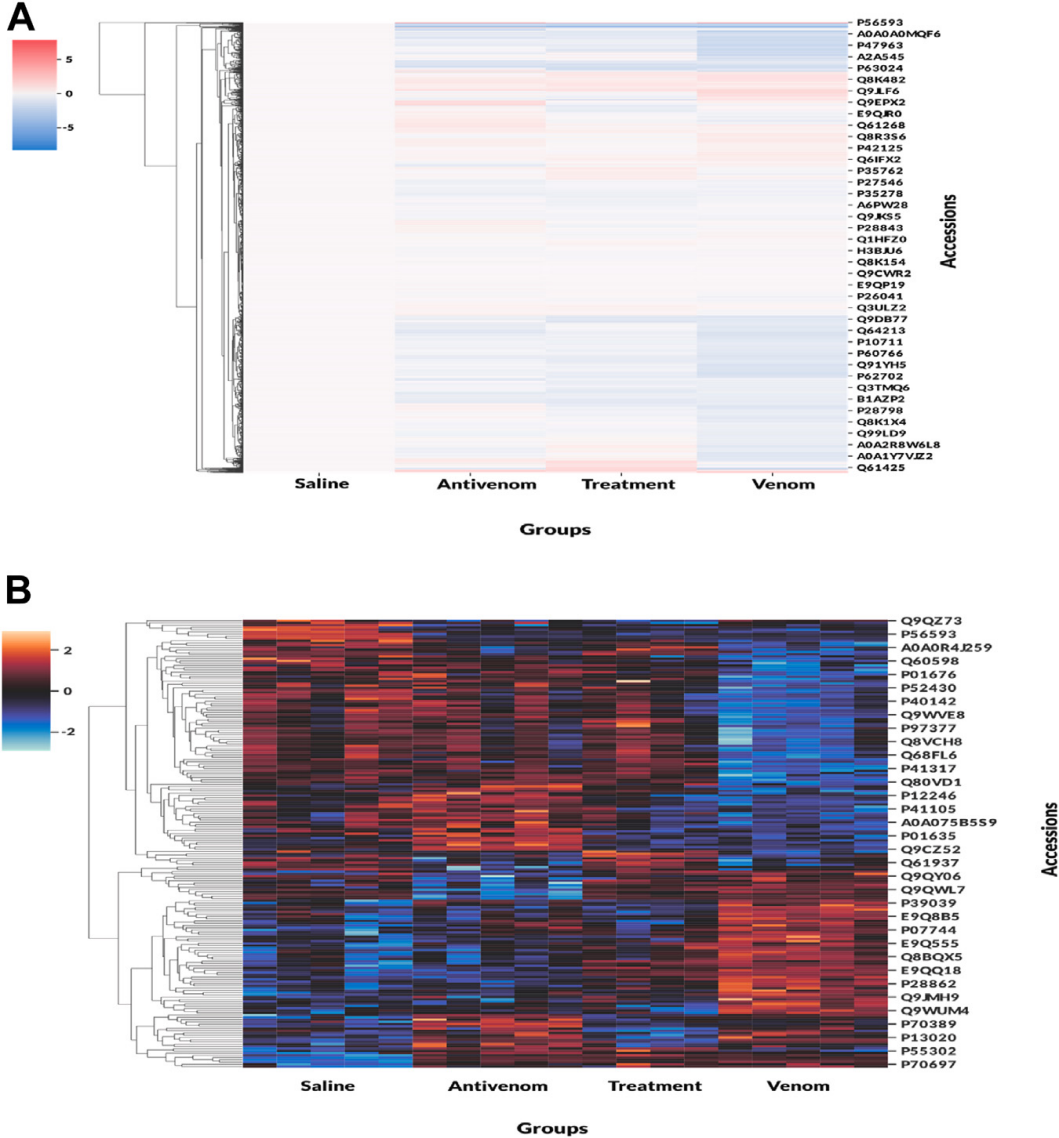
**Heatmap comparative analysis of protein abundance variations across different conditions.***A*, heatmap of identified proteins; comparing log ratios with saline condition as reference across 2100 proteins. *B*, heatmap of top proteins identified by ANOVA; comparing z-standardized protein abundance across conditions.

**Fig. 4:**
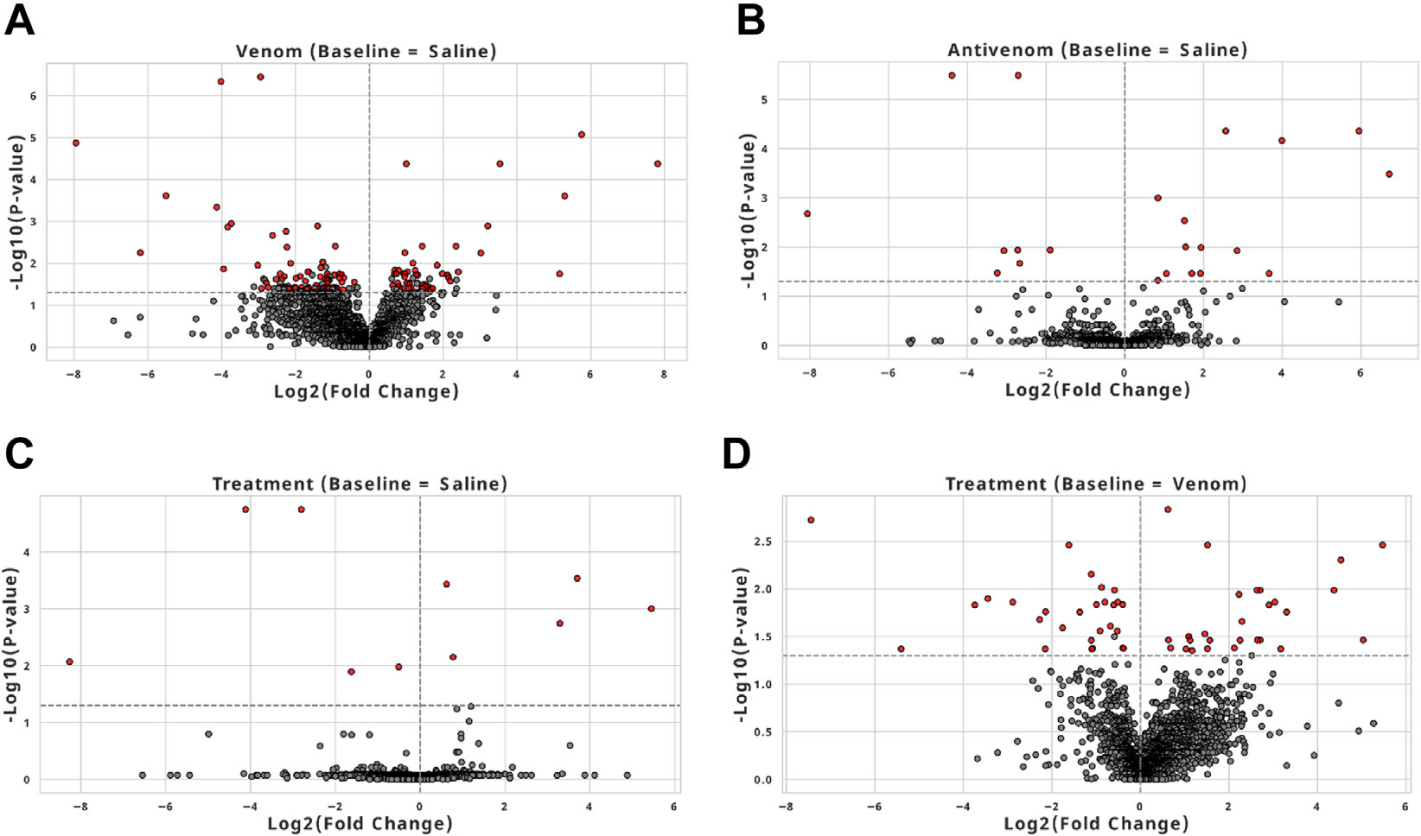
**Differential protein expression in volcano plots**. *A*, significantly expressed proteins (in *red*) when comparing venom *versus* saline conditions, those positioned to the *right* are upregulated while those positioned to the *left* are downregulated. All the significant proteins are listed in the Perseus out and rows containing the top twenty proteins are highlighted in *red* (supplemental Material S1). *B*, significantly expressed proteins (in *red*) when comparing antivenom *versus* saline conditions, those positioned to the *right* are upregulated while those positioned to the *left* are downregulated. *C*, significantly expressed proteins (in *red*) when comparing treatment *versus* saline conditions, those positioned to the *right* are upregulated while those positioned to the *left* are downregulated. *D*, a notable observation is the higher concentration of significant proteins (those positioned toward the top) when comparing the treatment to the venom baseline. This pronounced difference underscores the therapeutic efficacy of the antivenom in neutralizing venom effects.

We compared the protein Log2 expression ratios (saline, venom, treatment, and antivenom), which allowed us to explore the snake venom effects/responses on the mouse EVs, allowing for a systems-level understanding of snakebite pathophysiology *via* high abundance (log2 ratio >0) or low abundance (log2 ratio <0) of the identified proteins, which were mapped into mouse genes using Perseus (supplemental Material S1). We visualize the protein expression through heatmaps of normalized protein abundances; we observe different expressions when we compare log2 ratios of saline condition across conditions and proteins identified through ANOVA comparing z-standardization abundance across conditions (Fig. 3, *A* and *B*). For a more global visualization of the quantitation results, the volcano plots, using a *t* test with the saline group as the master control, contained quantitative comparison analyses of the plasma EV proteomes between venom, antivenom, and treatment conditions. Proteins highlighted in red meet three stringent criteria: significance in the *t* test, identification as significant in permutation shuffle, and achieving an accuracy greater than 0.8 in logistic regression (Fig. 4, *A*–*D*). Those in gray are significant based solely on the *t* test. The farther they shift to the left or right, the greater their deviation from the baseline (Fig. 4, *A*–*D*). This analysis revealed that 216 proteins were identified to be significantly downregulated (Fig. 4*A*, Blue), and 119 were significantly upregulated (Fig. 4*A*, Red) in the venom-specific condition. It was important to identify the nonvenom-related significant proteins or antivenom background, as shown in the antivenom plot (Fig. 4B). These proteins were either identified to be antivenom-specific (orange), shared between the venom/antivenom groups (black), or shared between all conditions (gray) (Fig. 4, *B* and *C*). Finally, a comparison of the venom condition against the treatment condition shows that 87% (293 of 341) of the proteins cross the significance threshold, suggesting significant neutralization of venom responses (Fig. 4 D) by antivenom.

### Regulated Biomarkers in Venom Compared with Nonvenom Conditions

**Fig. 5:**
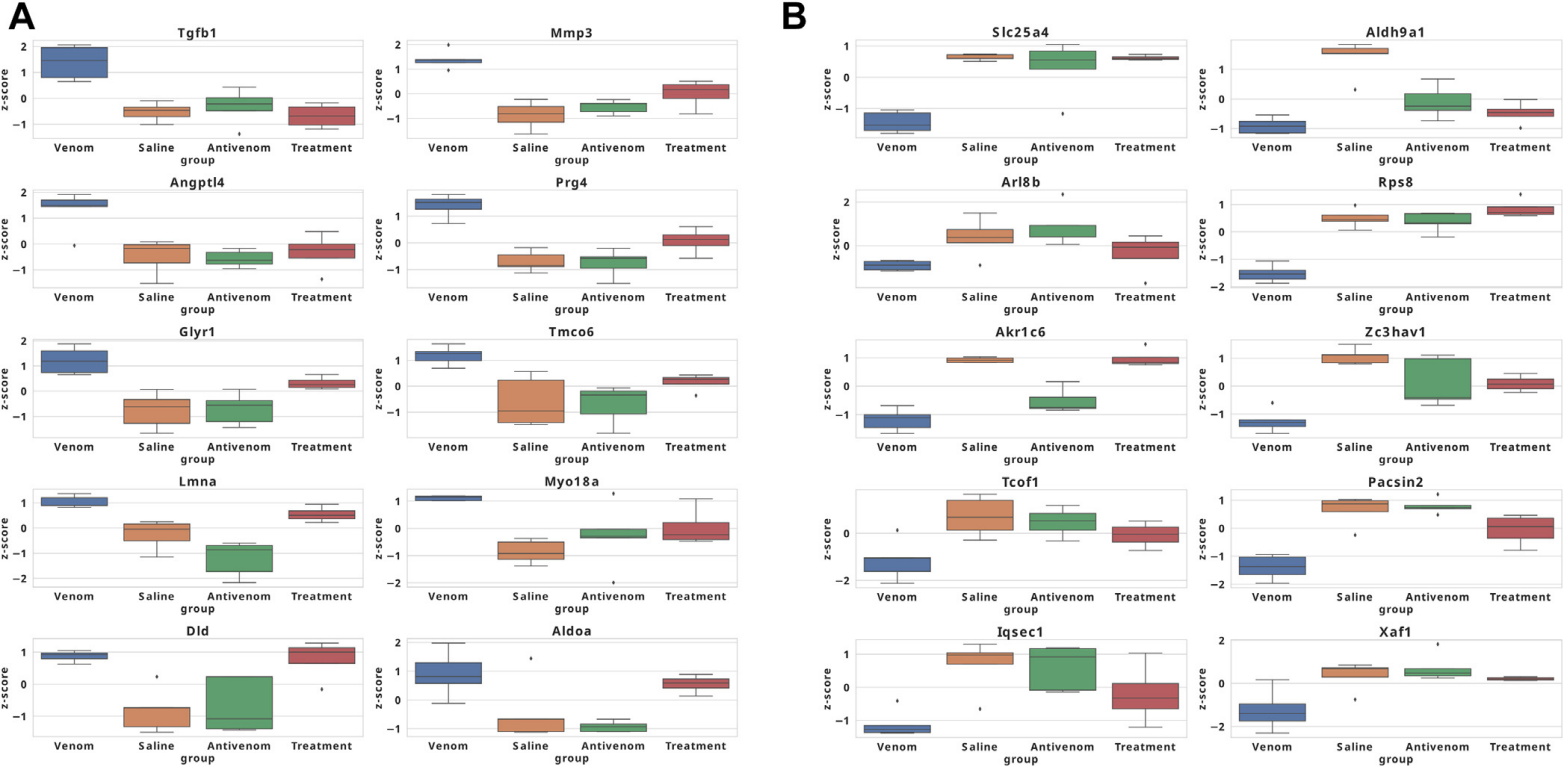
**Abundance of the top 10 upregulated proteins**. *A*, Z-standardized protein abundance of the top 10 upregulated proteins with the most significant (BH-adjusted *p* < 0.05) fold change between venom and nonvenom conditions. *B*, Z-standardized protein abundance of the top 10 downregulated proteins with the most significant (BH-adjusted *p* < 0.05) fold change between venom and nonvenom conditions.

We focused on the twenty most promising venom biomarker representatives that exhibited significant shifts in protein abundance levels between venom and nonvenom conditions when evaluated using log2 fold-change. Eighteen (18/20) of these proteins had more than two peptide hits for identification. The variations were graphically represented in linear box-and-whiskers plots (Fig. 5). Tgfb1 (P04202; Transforming growth factor beta-1 proprotein), Mmp3 (P28862; Stromelysin-1), Angptl4 (Q9Z1P8; Angiopoietin-related protein 4), Prg4 (E9QQ18; Proteoglycan 4), Glyr1 (D3YYT1; Glyoxylate reductase 1 homolog), Tmco6 (Q8BQX5; Transmembrane and coiled-coil domain-containing protein 6), Lmna (P48678; Prelamin-A/C), Myo18a (Q9JMH9; Unconventional myosin-XVIIIa), Dld (O08749; Dihydrolipoyl dehydrogenase, mitochondrial), Aldoa (P05064; Fructose-bisphosphate aldolase A) were significantly upregulated; however, we observed some upregulated biomarkers that did not significantly shift toward saline condition upon treatment, the most consistent among them are Dld and Aldoa (Fig. 5A). Slc25a4 (P48962; ADP/ATP translocase 1), Aldh9a1 (Q9JLJ2; 4-trimethylaminobutyraldehyde dehydrogenase), Arl8b (Q9CQW2; ADP-ribosylation factor-like protein 8B), Rps8 (P62242; 40S ribosomal protein S8), Akr1c6 (P70694; Estradiol 17 beta-dehydrogenase 5), Zc3hav1 (D3Z5I1; Zinc finger CCCH-type antiviral protein 1), Tcof1 (O08784; Treacle protein), Pacsin2 (Q9WVE8; Protein kinase C and casein kinase substrate in neurons protein 2), Iqsec1 (Q8R0S2; IQ motif and SEC7 domain-containing protein 1), Xaf1 (Q5NBU8; XIAP-associated factor 1) were significantly downregulated (Fig. 5*B*).

### Regulated Biomarkers in Venom Compared with Treatment Condition

**Fig. 6:**
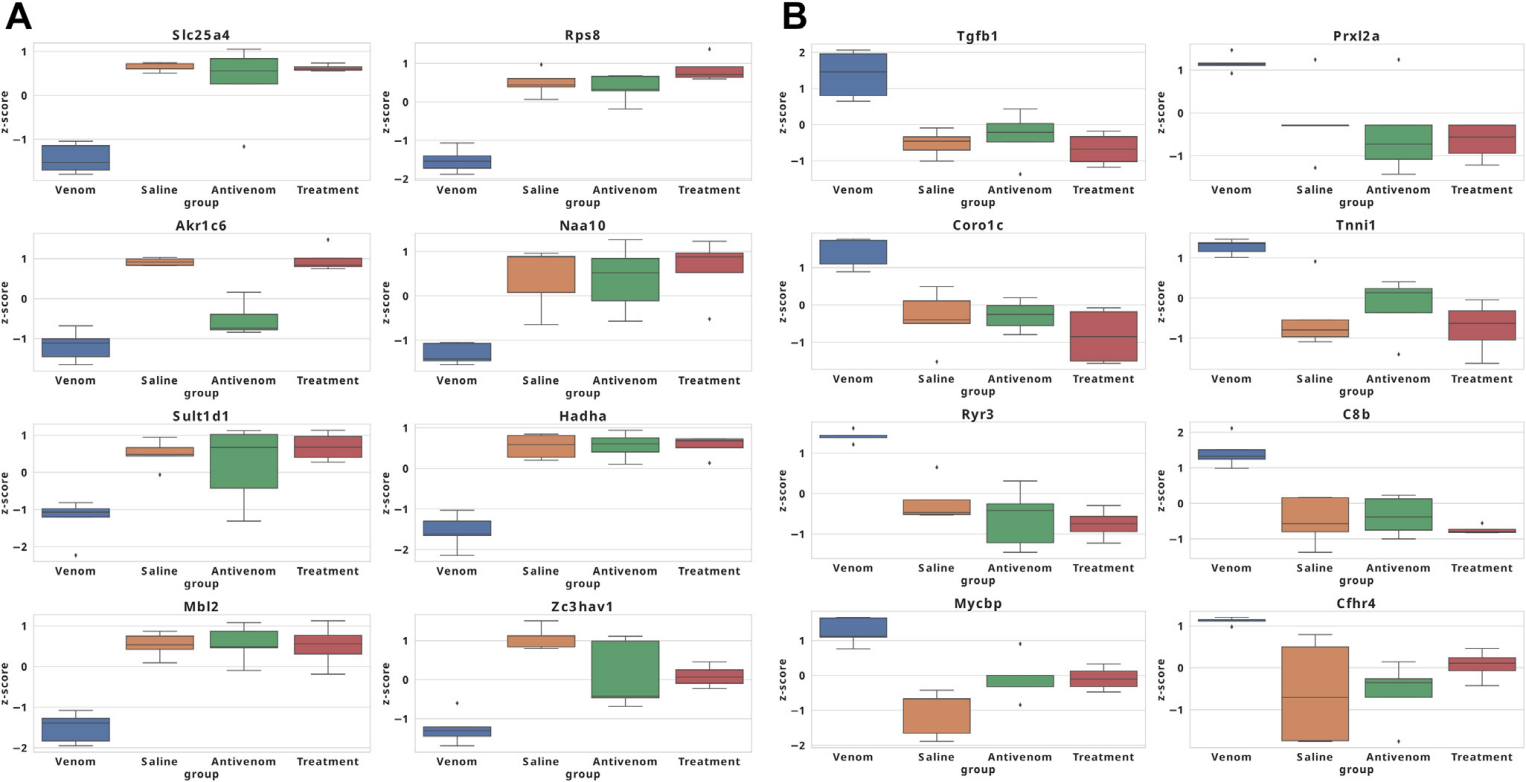
**Abundance of the top 10 downregulated proteins**. *A*, eight downregulated proteins sensitive to antivenom treatment. The depicted eight proteins are identified from the overlap of the top 50 proteins most significantly downregulated in the venom condition and those showing significant upregulation upon treatment. *B*, top eight upregulated proteins sensitive to antivenom treatment. The selected eight proteins are identified from the overlap of the top 50 proteins most significantly upregulated in the venom condition and those showing significant downregulation upon treatment.

Finally, we use linear box-and-whiskers plots to represent the eight downregulated and eight upregulated proteins significantly sensitive to antivenom treatment (Fig. 6). Slc25a4 (P48962; ADP/ATP translocase 1), Rps8 (P62242; 40S ribosomal protein S8), Akr1c6 (P70694; Estradiol 17 beta-dehydrogenase 5), Naa10 (B1AUY8; N-alpha-acetyltransferase 10), Sult1d1 (Q3UZZ6; Sulfotransferase 1 family member D1), Hadha (Q8BMS1, Trifunctional enzyme subunit alpha, mitochondrial), mannose-binding lectin 2 (Mbl2) (P41317; Mannose-binding protein C), Zc3hav1 (D3Z5I1; Zinc finger CCCH-type antiviral protein 1) were significantly downregulated in abundance levels in the venom condition and were restored after the antivenom therapy (Fig. 6*A*). Likewise, Tgfb1 (P04202; Transforming growth factor beta-1 proprotein), Prxl2a (Q9CYH2; Peroxiredoxin-like 2A) Coro1c (Q9WUM4; Coronin-1C), Tnni1 (Q9WUZ5; Troponin I, slow skeletal muscle), Ryr3 (A2AGL3; Ryanodine receptor 3), C8b (Q8BH 35; Complement component C8 beta chain), Mycbp (Q9EQS3; c-Myc-binding protein OS = *M. musculus*) and Cfhr4 (E9PUM5; Complement factor H-related 4), were significantly upregulated in the venom condition (Fig. 6*B*). These proteins that are sensitive to antivenom treatment affect some interesting biological processes (supplemental Material S4).

### Significant Biomarkers Sensitive to the System’s Reaction to Antivenom

Due to the importance of hypersensitivity reactions in antivenom treatment of SBE, we use the antivenom-alone condition as the main control and the protein Log2 expression ratios in Perseus (saline, venom, and treatment). This has allowed us to identify and quantify significant proteins that may be upregulated or downregulated in reaction to the administration of antivenom into the biological system. On comparing the saline condition with the antivenom condition, we identified the top five significant downregulated proteins (Gpx4, Sub1, Prm2, Cyp2a12, and Fndc1) and the top five significant upregulated proteins (Igfals, Rack1, Pafah1b1, Fam107b, and Igfbp5). In addition, we compared the treatment condition (venom-alone, venom-antivenom, and antivenom-alone) *versus* the saline condition. We interestingly found overlapping proteins: five upregulated and five downregulated proteins specific to the system’s reaction to both antivenom and venom conditions (Table 1). We visualized the data through volcano plots to see the level of the significant proteins (Fig. 4, *A*–*D* and supplemental Material S5).

**Table 1:** Top 10 proteins expression hypersensitive to antivenom compared to the saline condition

Accession	Gene name	Description
Upregulated proteins		
P70389	Igfals	Insulin-like growth factor-binding protein complex acid labile subunit
P63005	Pafah1b1	Platelet-activating factor acetyl hydrolase IB subunit beta OS
P68040	Rack1	Receptor of activated protein C kinase 1 OS
A0A2I3BR29	Fam107b	Protein FAM107B
Q07079	Igfbp5	Insulin-like growth factor-binding protein 5
Downregulated proteins		
O70325	Gpx4	Phospholipid hydroperoxide glutathione peroxidase
P11031	Sub1	Activated RNA polymerase II transcriptional coactivator p15 OS
P07978	Prm2	Protamine-2 OS
P56593	Cyp2a12	Cytochrome P450 2A12 OS
A0A6I8MWX0	Fndc1	Fibronectin type III domain-containing 1

### Biological and Network Analysis

**Fig. 7:**
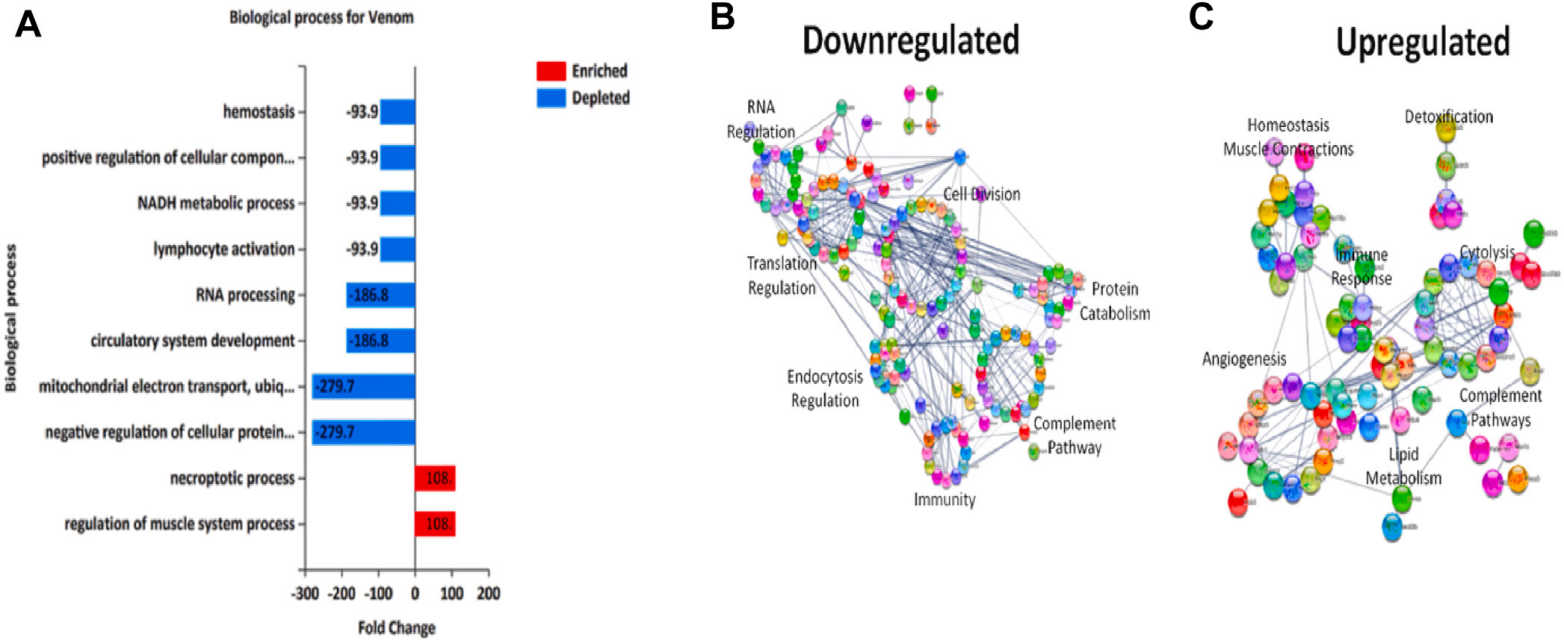
**Biological and pathway responses.***A*, the magnitude of impact on the enriched (*red*) and depleted (*blue*) biological processes specific to envenoming. *B* and *C*, STRING analysis of downregulated/upregulated pathways, respectively.

After these proteins were identified and extracted, we then performed enrichment analysis using DAVID to identify biologically relevant processes that would provide meaningful associations to the extensive proteomics data. Moreover, these bioinformatics analyses allowed for the elucidation of physiological responses sensitive to envenoming directly or indirectly 24 h post intramuscular administration (Fig. 7). The most prominently downregulated pathways were the hemostatic, metabolic, protein processing, and mitochondrial processes, whereas the most prominent upregulated biological processes were in cytolysis/necrosis, and muscle system regulation (Fig. 7*A*). To further confirm these observations, we used FunRich as a secondary bioinformatics analytics tool to validate our initial findings and create a circular string analysis. DAVID analysis (supplemental Material S6) supports the magnitudes of significantly enriched proteins based on their reported biological activity found in (Fig. 7 *A–C*). This is further supported by what was found in our previous study (48).

### Prediction Accuracy for the Top Significant Biomarkers

**Fig. 8:**
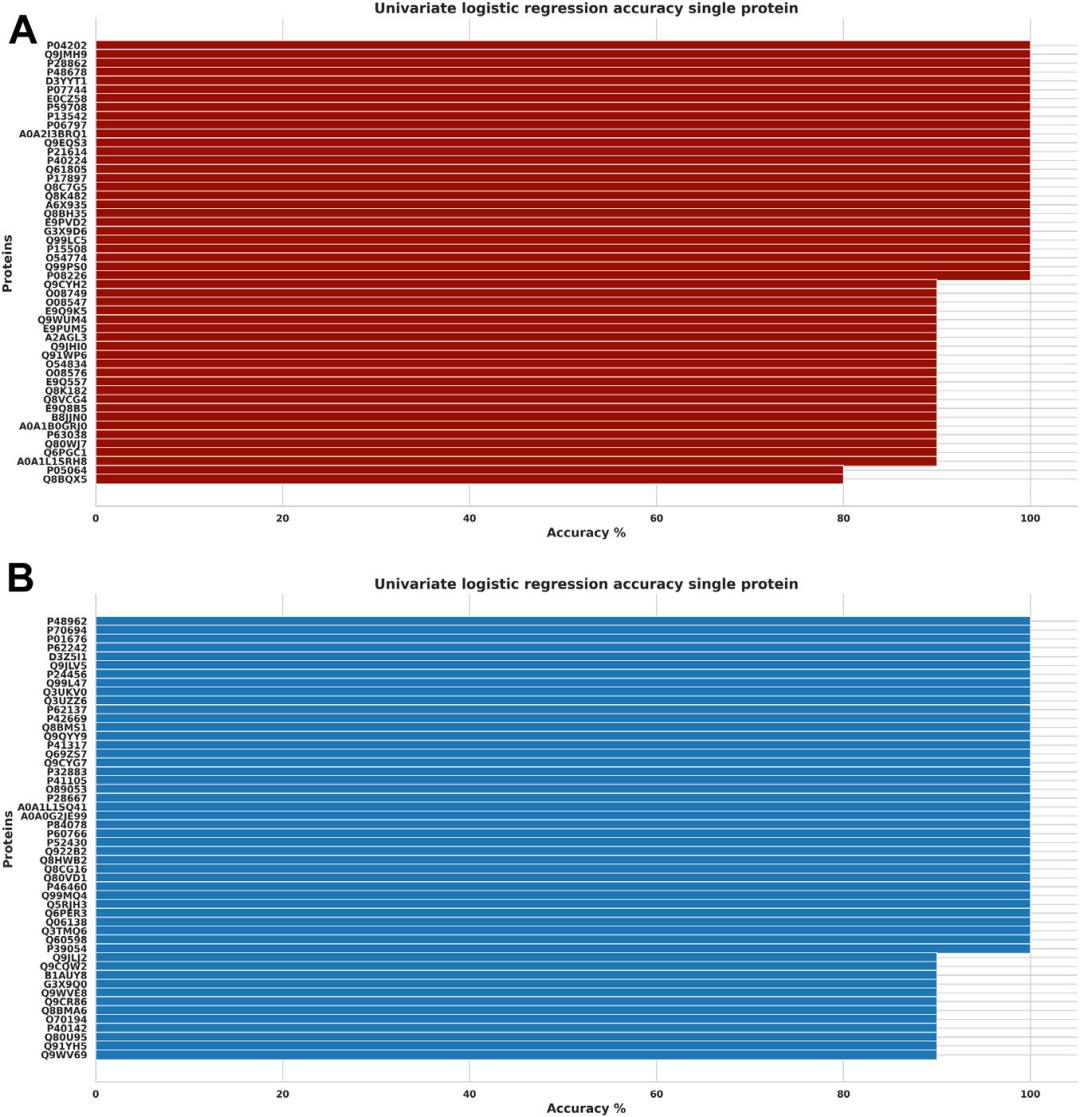
**Univariate logistic regression accuracy scores (Top 50) for significant venom biomarkers.***A*, across upregulated venom biomarkers in discriminating between venom and saline conditions; *B*, across downregulated venom biomarkers in discriminating between venom and saline conditions.

**Fig. 9:**
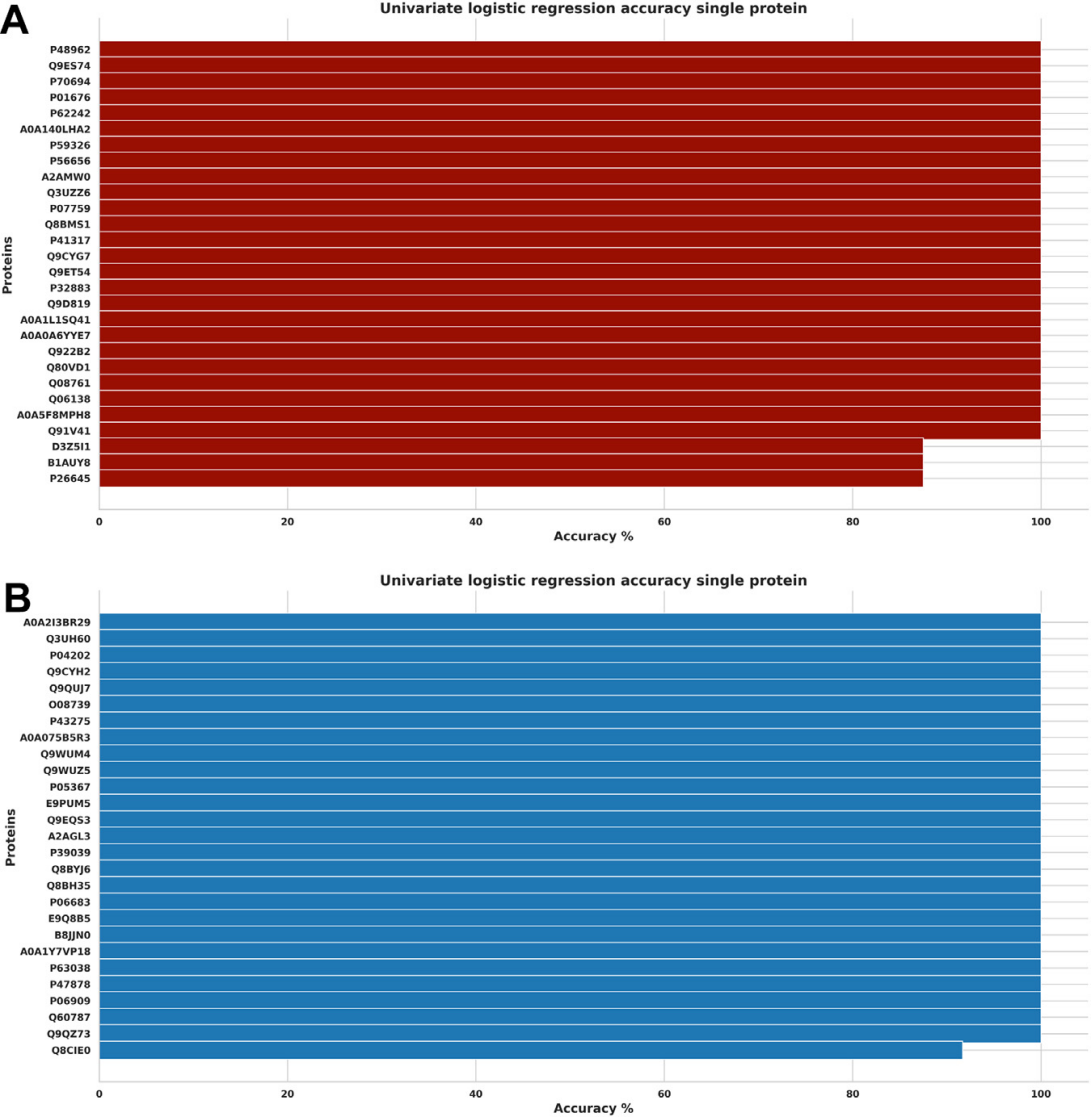
**Univariate logistic regression accuracy scores (top 55) for significant venom biomarkers.***A*, across upregulated treatment biomarkers in discriminating between venom and treatment conditions; *B*, across downregulated treatment biomarkers in discriminating between venom and treatment conditions.

**Fig. 10:**
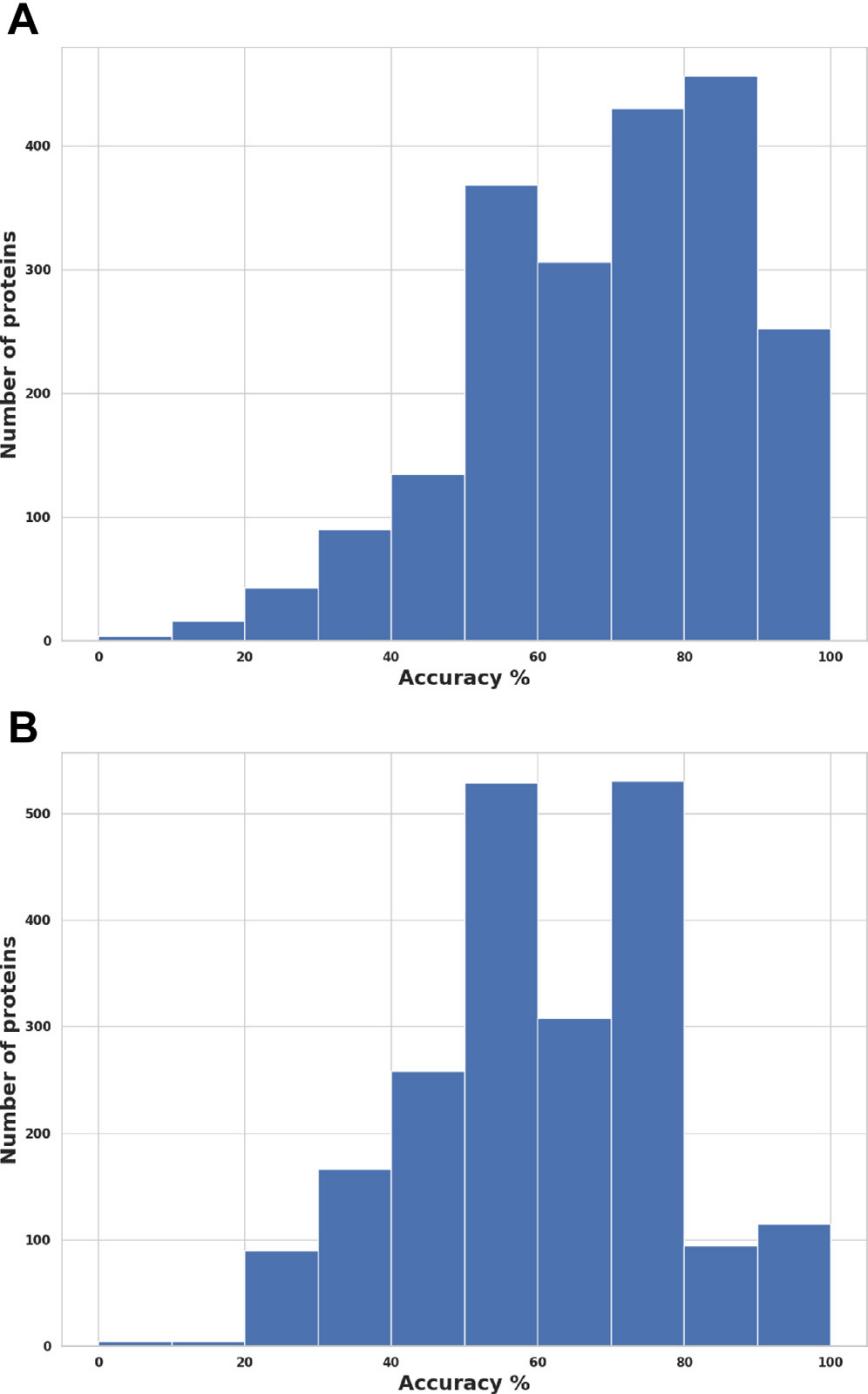
**Univariate logistic regression accuracy scores in discrimination analysis**. *A*, distribution of accuracy scores (using individual protein logistic regression) for discriminating between venom and saline conditions; *B*, similar distribution for discriminating between venom and treatment conditions.

**Fig. 11:**
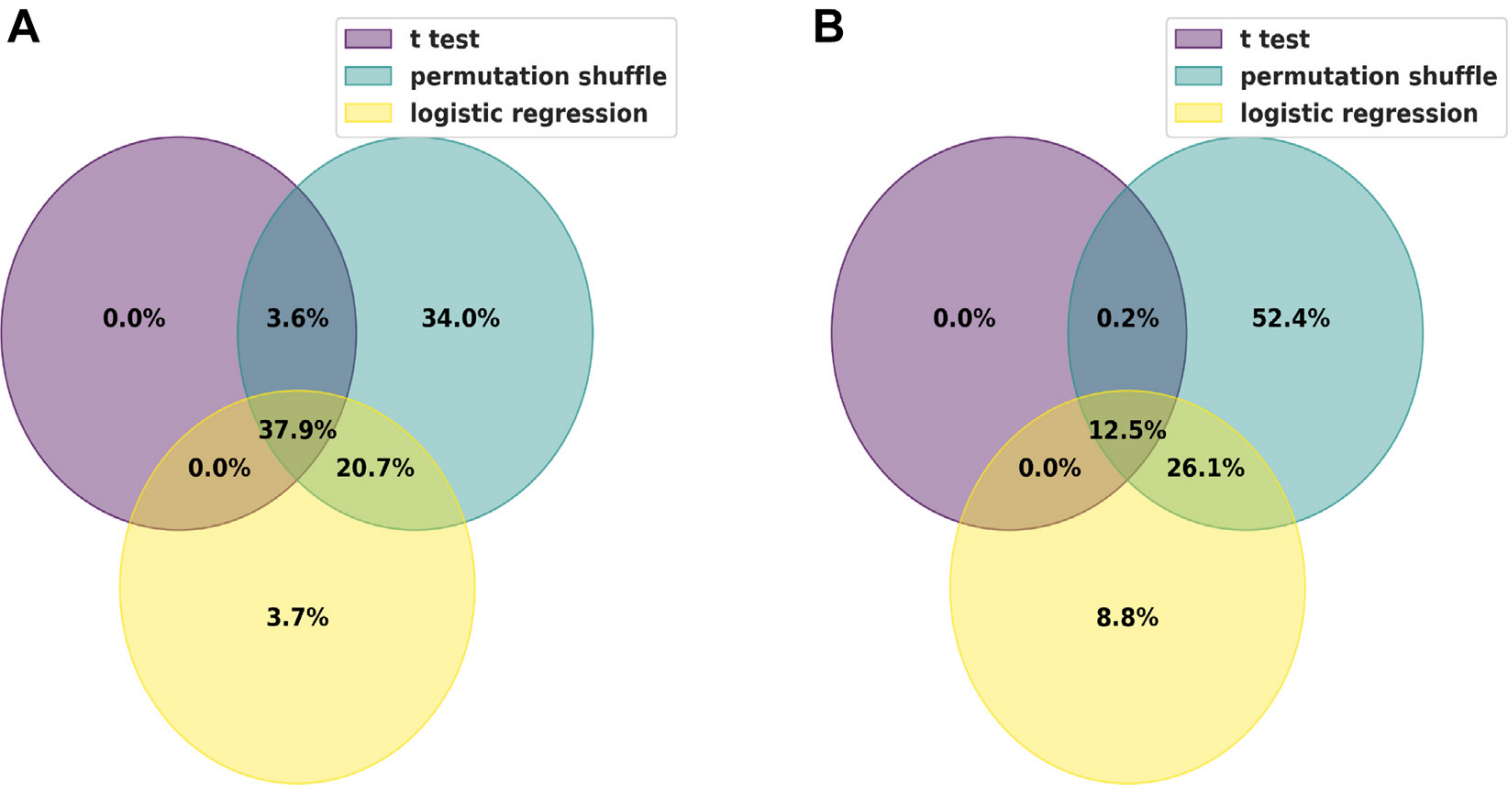
**Venn diagrams highlighting overlapping significant proteins**. *A*, the intersection of proteins found with significant differences between saline and antivenom conditions using *t* test (*B*–*H* adjusted *p* < 0.05), permutation shuffle (*p* < 0.05 from 1k permutations), and logistic regression (accuracy >80%). *B*, similar intersections between venom and treatment conditions.

In discriminating significant proteins for venom and saline conditions using univariate logistic regression, the accuracy scores indicate an excellent prediction model, with all the upregulated and downregulated proteins showing an accuracy above 70%. Twenty-four upregulated proteins scored 100%, 24 scored 90%, and 2 scored 80% accuracy; however, 38 downregulated proteins scored 100%, while 12 proteins scored 90% (Fig. 8, *A* and *B*). Additionally, we obtained excellent prediction accuracy for venom and treatment conditions, with 25 upregulated proteins scoring 100% while three scored 90% accuracy. Similarly, 26 downregulated proteins scored 100%, while 1 protein scored 90% accuracy (Fig. 9, *A* and *B*). Furthermore, we found a similar distribution of accuracy scores (individual proteins) when discriminating between venom *versus* saline conditions and venom *versus* treatment conditions (Fig. 10, *A* and *B*). Finally, all proteins identified as significant through the *t* test are also captured by the other two methods, reinforcing the accuracy of the findings, and minimizing the risk of including false positives (Fig. 11). We also found 37.9% of intersecting proteins with more than 80% accuracy scores and significant differences (*t* test, permutation shuffle, and logistics regression) when comparing saline versus antivenom (Figs. 11*A*) and 12.3% with similar accuracy and significance when comparing venom *versus* treatment conditions (Fig. 11*B*).

## Discussion

The objective of this study was to analyze the change in proteomic abundances, identify novel biomarkers, and predict promising biomarkers in murine plasma EV in response to envenomation. This was accomplished by combining animal experiments, mass spectrometry, and various bioinformatic approaches to detect the fluctuation in global systemic signatures from various conditions. *C. atrox* is medically relevant regarding the typical skin/tissue/muscle necrosis, and hemostatic disturbances that often lead to sequelae (2, 51). We have previously used liquid chromatography mass spectrometry to identify the highly abundant L-amino acid oxidases, SVMP, snake venom serine proteases, and phospholipases A_2_s (PLA_2_) (making up 6%, 31%, 21%, and 11% of the whole venom, respectively) as the protein toxins most responsible for these effects (48).

Viperid and Crotalid venoms induce both local and systemic effects, directly or indirectly, due to the action of the toxins on cells, extracellular matrix, and plasma. Moreover, these venoms can activate inflammatory reactions and release DAMPs from the surrounding cells and tissues (22, 52), which can then activate and alter other downstream physiological processes. These mechanisms in SBE are poorly defined and require more system approaches to identify the cellular responses and pathways involved. Circulating EVs have been reported in various pathophysiological conditions by the analysis of various cell-derived EVs in the systemic circulation (red blood cells, leukocytes, platelets, and endothelial cells) (42). The snakebite envenoming's effects cause significant coagulopathy, and changes in the normal processes of hemostasis and thrombosis are widely recognized (53, 54).

We mimic a real-life scenario of snakebite by designing a rescue assay (n = 5 for each of the four conditions) to analyze EVs from a plasma extract in the systemic circulation of BALB/c mice after *C. atrox* envenoming as well as its comparison with antivenom treatment. Herein, we have investigated the pathology induced by *C. atrox* venom, quantified and identified the changes in the plasma EVs as readouts of snake venom biomarkers revealed by our proteomic analyses of murine plasma EVs post envenomation. We found reliable distribution patterns and correlations between sample pairs for protein abundance levels for the 19 samples treated with venom, saline, and antivenom (supplemental Materials S2 and S3). Our findings show significant perturbations in several metabolic pathways and uncover a possible snake venom-specific mechanism to maintain homeostasis, thus contributing to a better understanding of this poorly studied aspect of envenoming. Moreover, the use of antivenom in our model provides system-level details of specific biomarkers that can be modulated by therapeutics and/or useful in the early detection of hypersensitivity reactions to antivenom.

We found that tricarboxylic acid (TCA)/NADH metabolism, endocytosis, RNA regulation, and other biological processes were downregulated upon *C. atrox* venom treatment (Fig. 3). The upregulated responses included lipid metabolism, immune response, and P450/detoxification. We focused on a selection of proteins that exhibited significant changes, identifying key representatives that may serve as promising biomarkers. The relative abundances of these proteins across different experimental conditions—saline, venom, antivenom, and treatment group—were quantitatively represented using linear box-and-whiskers plots (Figs. 5 and 6). Interestingly, we could not find a protein-by-protein coincidence among the expressed proteins between our current study and the previous one (48). However, we found both high and low changes in protein abundance that are involved in similar pathways to the ones we saw in our previous study, these include cytochrome P450 involvement, metabolic processes, lipid metabolism, and acute phase inflammation. The variability in protein abundance between our previous and current study could be a result of the alteration of the experimental design from our previous study (48); this is in our attempt to mimic a real-life snakebite scenario, making the current design more suitable for biomarker identification.

Indeed, no prior studies substantiated the scientific rationale for these targets as possible SBE biomarkers, but these could be promising novel diagnostic markers for further clinical validation. The six most consistently significant biomarkers sensitive to treatment were Mbl2, Hadha, and Slc25a4 (downregulated), and Ryr3, C8B, and Prxl2a (upregulated). Mbl2 is an innate immune defense protein secreted by the liver as part of the acute-phase response. Mbl2 ligands are expressed by a wide range of microorganisms, and binding of the protein results in opsonization and complement activation (55, 56). The mitochondrial trifunctional protein Hadha beta subunits are a part of the heterocomplex that contains 4 alpha and 4 beta subunits and catalyzes three steps in mitochondrial beta-oxidation of fatty acids, including the long-chain 3-hydroxyacyl-CoA dehydrogenase step (57, 58). The ATP-Mg solute carrier Slc25a4 transports adenine nucleotides to the matrix of the mitochondria in response to cytosolic Ca^2+^ (59). The Ryr3 gene encodes ryanodine receptor-3, a family of intracellular calcium ion release channels responsible for releasing Ca^2+^ from intracellular stores following stimulation (60). C8B is a complement component 8 (C8) protein subunit. C8 is a component of the membrane attack complex that mediates cell lysis and initiates the complex's membrane penetration (60, 61). It plays a key role in the formation of the membrane attack complex, which is an important antimicrobial immune effect (62). Studies showed that sC5b-9, C3bc, C4bc, and C3bBbP were elevated in acute heart failure patients (63). Prxl2a is predicted to enable antioxidant activity and is predicted to be involved in the regulation of osteoclast differentiation. It is located in the mitochondrion and expressed in several structures, including the alimentary and urinary systems (64).

*C. atrox* venom-induced perturbations in the TCA/NADH, lipid metabolism, cytolytic, hemostatic, and immune responses in mouse plasma EVs. Our DAVID analysis revealed 19 markers (Gene names: Bdh1, Adh4, Akr1c6, Angptl3, Angptl4, Cyp2c39, Enpp2, Ggps1, Gpx1, Gpx4, Hadha, Hsd17b11, Pam, Plin1, Plcb3, Pafah1b1, Prkaa1, Rnf213, and Sult1d1) associated with lipid metabolism (supplemental Material S6), which presents an interesting observation. Injury due to SBE has been associated with metabolic changes, lipolysis, and fatty acid mobilization, changes in amino acid levels, nucleotide breakdown, increased glycolysis, plasma elevation of TCA intermediates, and accumulation of ketone bodies, among other alterations. We have previously identified several biomarkers in our models, showing that metabolic changes in mice are due to injury (48). Similar results were found in a proteomic study of critically injured thoracotomy patients (65). They identified trauma-dependent metabolic signatures, which support a state of hypercatabolism, lipolysis, and fatty acid oxidation, accumulation of ketone bodies, proteolysis, and nucleoside breakdown. This suggested a plausible hypothesis for the body’s need for carbon and nitrogen to compensate for trauma-induced energy consumption and negative nitrogen balance (65, 66).

To further support this observation, a study by Wase et al. (67) using metabolomics and lipidomic approaches to identify biomarkers from plasma has pointed to alterations in metabolic pathways associated with amino acid synthesis, TCA cycle, aminoacyl tRNA synthesis, and amino acid biosynthesis. Oxidative stress makers of cortisol and heme were also found and suggest a pattern of traumatic injuries [(68, 69)]. Based on these results, they speculated that snake venoms possess mediators of permeability, which could promote disruption of cells in endothelium, blood–brain barrier, and the intestinal barrier during viperid envenoming, promoting changes to metabolites. However, more studies are warranted using proteomics, lipidomics, and metabolomics to gain further insight into the exposome of snake venom. In snakebite envenoming, the changes in some metabolites could be due to the cellular apoptosis from snake venom serine proteases, SVMP, or phospholipases A_2_s on tissue and blood substrates (70, 71).

The use of antivenom is the mainstay approach to managing SBE; however, it can induce severe acute or delayed hypersensitivity reactions in many victims of SBE making early detection and resolution of these adverse reactions an important goal for clinicians involved in the management of SBE (72). Interestingly, there are reports of biomarkers linked to certain hypersensitivity reactions to drugs (73, 74), including chemotherapeutics (75) and snake antivenoms (76). One of our experimental conditions received antivenom alone, and we observed certain significant perturbations in some of the proteins (Gpx4, Sub1, Prm2, Cyp2a12, Fndc1, Igfals, Rack1, Pafah1b1, Fam107b, and Igfbp5) identified in this condition in comparison to the saline group. Additionally, some of the significant proteins (Gpx4, Sub1, Prm2, Cyp2a12, Pafah1b1, and Rack1) were also expressed in the treatment group post administration of antivenom (Table 1). These changes in the abundance of the proteins could be a result of the body’s immune response to the antivenom, which usually leads to either late or delayed hypersensitivity reactions, as earlier reported (77, 78, 79, 80).

Prediction of these biomarkers using bioinformatics tools holds immense potential for early detection and management of SBE and antivenom-related reactions, ultimately reducing morbidity and mortality. This could also provide a scientific basis for antivenom design, enhancing its effectiveness in neutralizing venom toxins and early detection and attenuation of severe hypersensitivity reactions. This approach offers valuable insights into the proteomic responses to both venom and antivenom. *C. atrox* envenoming produced a complex multisystem response in the changes to plasma EV protein abundance in mice. As the studies continue to progress, these common changes in biological responses may represent the typical reaction of the organism to traumatic injuries. Understanding the role that these biomarkers play directly or indirectly in the envenoming process is still unknown. However, this study expands upon the need for more diagnostic tools to improve the management of this neglected tropical disease.

More studies are needed to test other snake venoms using different doses of venom and different antivenoms early to improve our understanding of the systemic pathology and the acute responses after envenoming to identify and validate more consistent biomarkers and establish better baseline antivenom responses. The identified markers that were consistently and significantly sensitive to antivenom treatment were Slc25a4, Rps8, Akr1c6, Naa10, Sult1d1, Hadha, Mbl2, Zc3hav, Tgfb1, Prxl2a, Coro1c, Tnni1, Ryr3, C8b, Mycbp, and Cfhr4 (Fig. 6, *A* and *B* and supplemental Material S5). Interestingly, we observed proteins that were significantly perturbed in the venom condition but did not show a significant shift toward normal (saline condition) upon treatment with antivenom; the most significant among them are the upregulated Dld and Aldoa. These could be from pathogenic EVs indicating envenoming pathways different from those induced by snake venom major toxins that are sensitive to antivenom. We have seen clear similarities in protein distribution in our rescue approach which could be why we observed not a very sharp mirror overlap of the protein distribution pattern between the treatment condition and the saline condition (Fig. 2*A*). For testing antivenom candidates, a preincubation approach could be added which involves premixing venom and antidote before inoculating the experimental animals. There have been reports of significant differences in dose and response between the two preclinical antivenom treatment approaches, even among *Crotalus* species (81, 82). It is also advocated that testing therapeutics that are not antibody-based will be better done with a rescue experimental approach (81); however, the antivenom used in our study is a horse antibody-based generated antivenom. Thus, as the gold standard in preclinical testing of antibody-based antivenoms, the preincubation of venom and antivenom is designed to completely or significantly neutralize the toxic properties of venom toxins before they are introduced into the biological system (83).

Murine studies have shed light on venom-induced tissue damage mechanisms through proteomic analysis of venom exudates, it was found that *Bothrops asper* venom caused cytotoxicity, plasma exudation, extracellular matrix degradation, and membrane protein shedding, identifying biomarkers for myonecrosis and microvascular damage [(84), Rucavado] Similarly, exudates from wounds caused by *BaP1* (metalloproteinase) and *Mtx-I* (phospholipase A2) revealed toxin-specific effects where BaP1 degraded nonfibrillar collagens crucial for capillary stability, while Mtx-I caused fibrillar collagen type I, apolipoproteins, and fibronectin release, disrupting hemostasis [(85), Escalante]. Additionally, human studies by Macêdo *et al*. (86) and Smith *et al*. (87) analyzed venom effects in clinical cases. Macêdo *et al*. identified 805 proteins in blister fluids from patients bitten by *Daboia russelii*, *Hypnale hypnale*, and *Naja naja*, linking them to platelet degranulation, immune responses, complement activation, and coagulation (86). Smith *et al*. analyzed dried blood spots from a lethal rattlesnake (*Crotalus viridis viridis*) envenomation, detecting proteins associated with inflammation, coagulation disorders, and hypoxia (87). Human studies reflect clinical variability and venom reservoirs like blister fluid, while murine models provide controlled conditions for exploring venom mechanisms. However, analyzing EVs after envenomation reveals specific molecular changes but requires additional purification steps compared to analyzing biofluids like wound exudates, blood, or plasma. While biofluids provide immediate systemic responses, EVs reflect more focused cellular processes (88).

## Proposed Model of Venom and Antivenom Cellular Responses Mediated by Extracellular Vesicles

**Fig. 12:**
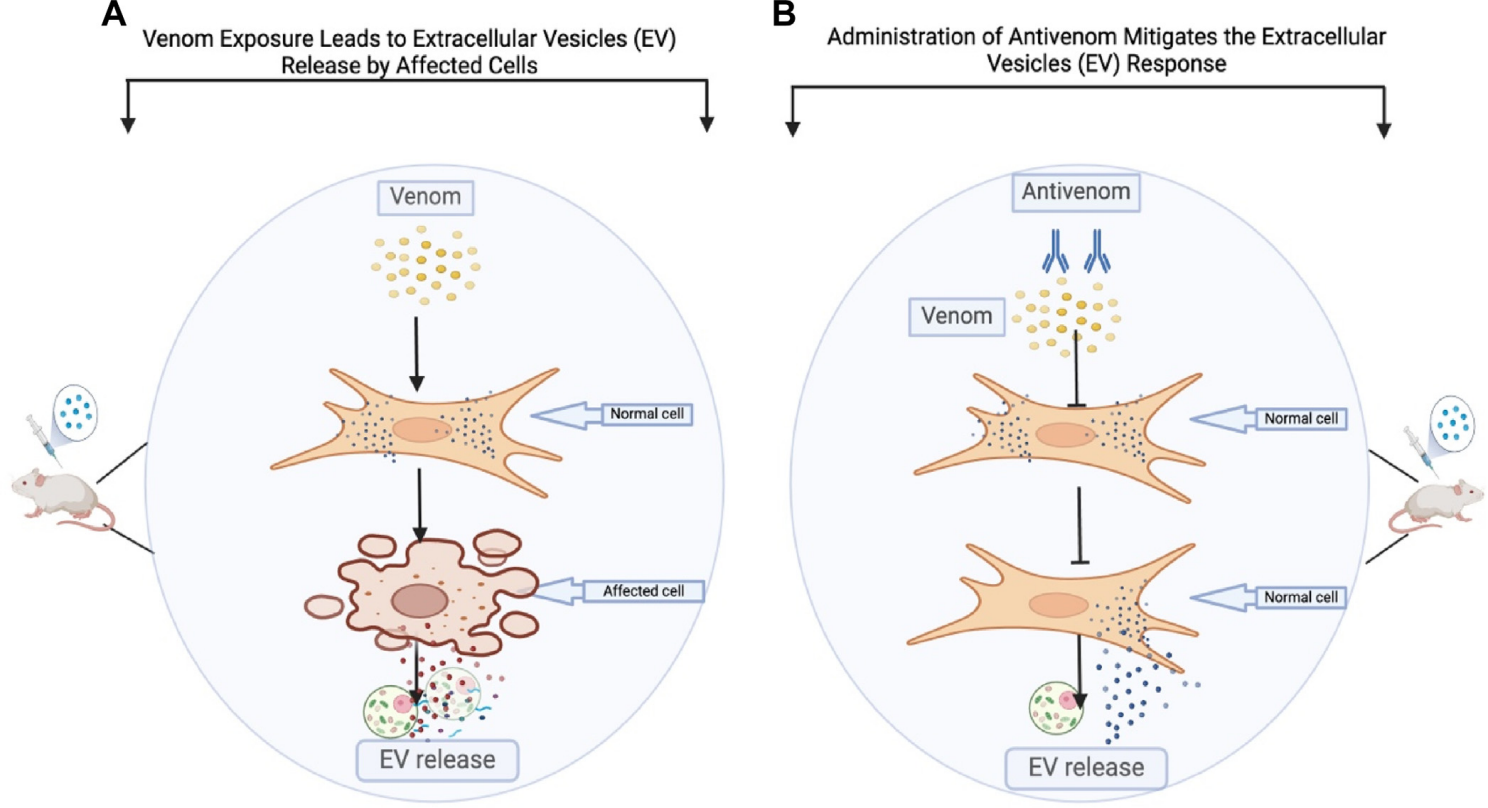
**Proposed model of venom and antivenom cellular responses mediated by extracellular vesicles**. *A*, cells exposed to venoms undergo cellular and molecular responses including necrosis and release of extracellular vesicles (exosomes) that communicate with other cells through their contents comprising proteins and nucleic acids. *B*, administration of antivenom post venom exposure leads to neutralization of venom before it causes significant cellular and molecular changes.

It is well established that when an organism is exposed to toxic substances, the affected/target cells release EVs that communicate with other cells to facilitate toxicity processes such as inflammation, cell damage, and other pathologies (89, 90, 91). The biological system’s EVs do this by transporting a cargo containing proteins, lipids, metabolites, and nucleic acids such as miRNAs, DNA, mRNAs, cytokines/chemokines, DAMPs, tissue factor, and caspases, which play roles in causing theses pathologies due to the initial toxicants exposure to the biological system. We exposed mice to the snake’s whole venom intramuscularly and then checked the plasma EV response through their protein cargo. Additionally, the administration of antivenom is directly targeted at neutralization of the venom *in vivo*. We proposed that the neutralization of the venom will prevent/reverse its toxic effect on the mice thereby preventing/mitigating the release of mice plasma EVs that communicate the system’s toxic processes, which we demonstrated by checking the changes in abundance of the mice plasma EV protein cargo using mass spectrometry-based proteomics (Fig. 12).

## Conclusions

These results provide a systems biology approach to studying the pathophysiological biomarkers and the exposome of toxicity in SBE by analyzing the contents of EVs extracted from blood plasma using mass spectrometry and bioinformatics. *C. atrox* venom caused significant perturbations in various physiological responses, such as altered changes in mitochondrial homeostasis, cell division, communication, metabolism, immunity, RNA regulation, cytolysis, and muscle contraction processes. The antivenom treatment showed the potential to neutralize most of the toxic effects by restoring the homeostatic physiology to near-control conditions (87% efficacy). We aim to explore and expand our methods to other snake venoms, isolated toxins, and antivenoms to help characterize venom toxicity and to evaluate therapeutic efficacy. Ultimately, we expect that our technology can be used to monitor the clinical effects of snakebites envenoming and antivenom treatment and reactions in patients, allowing for the identification of novel clinical diagnostics, prognostic, and predictive biomarkers.

## Data Availability

The study dataset containing raw files, peaks, results, and annotated spectra zip (the byonic was embedded into the Proteome Discoverer) is deposited in jPOST through ProteomeXchange with the following identifier: http://proteomecentral.proteomexchange.org/cgi/GetDataset?ID=PXD051784 and Link to the full dataset: https://repository.jpostdb.org/entry/JPST003057 (Username: auwal.bala@fud.edu.ng, Password: Auwalili). The bioinformatics output and other supporting data are provided as supplemental Materials S1–S6 within the manuscript.

## Supplemental data

This article contains supplemental data.

## Conflicts of Interest

A.I. is a principal at Tymora Analytical Operations, which developed the EVtrap technology.

## Supplementary Data

Suplimmental material 1

Suplimmentary File 2

Suplimmentary File 3

Suplimmentary File 4

Suplimmentary File 5

Suplimmentary File 6
