# Supplementary material for: Bioinformatics-Guided Identification and Quantification of Biomarkers of Crotalus atrox Envenoming and Its Neutralization by Antivenom
Source: Mol Cell Proteomics. 2025 Mar 25;24(5):100956. doi: 10.1016/j.mcpro.2025.100956 (PMC12140956; doi:10.1016/j.mcpro.2025.100956)
Supplement: Suplimmentary File 2 [file mmc2.docx]

Supplementary material 2: Comparative Log10 Abundance of Proteins Across Different Samples


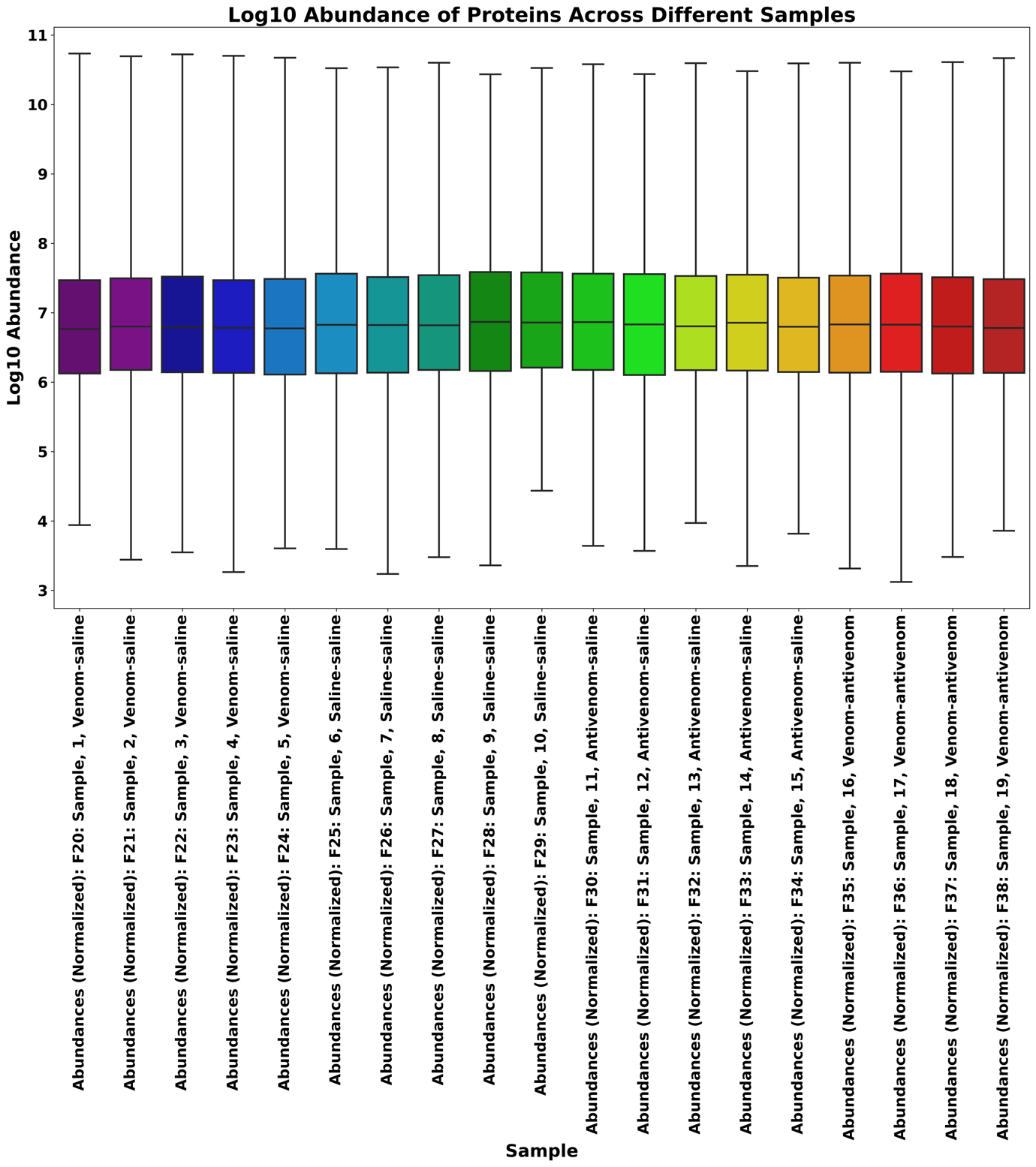


**S2: Comparative Log10-transformed protein abundance profiles in response to various treatments.**The box plots delineate the distribution of protein abundance levels for 19 samples treated with venom, saline, and antivenom. The central line in each box represents the median value, the box limits indicate the first (Q1) and third (Q3) quartiles, and the whiskers extend to 2.5 times the interquartile range (IQR) from the box limits, highlighting the range of the data. The samples are labeled with their respective treatments and identifiers (F20-F38). Data points illustrate the log10 abundance with minimum and maximum values, emphasizing the variability and spread within each treatment group.
