# Supplementary material for: Bioinformatics-Guided Identification and Quantification of Biomarkers of Crotalus atrox Envenoming and Its Neutralization by Antivenom
Source: Mol Cell Proteomics. 2025 Mar 25;24(5):100956. doi: 10.1016/j.mcpro.2025.100956 (PMC12140956; doi:10.1016/j.mcpro.2025.100956)
Supplement: Suplimmentary File 3 [file mmc3.docx]

Supplementary Material 3: Heatmap of Proteins Abundance Correlation Across Different Samples


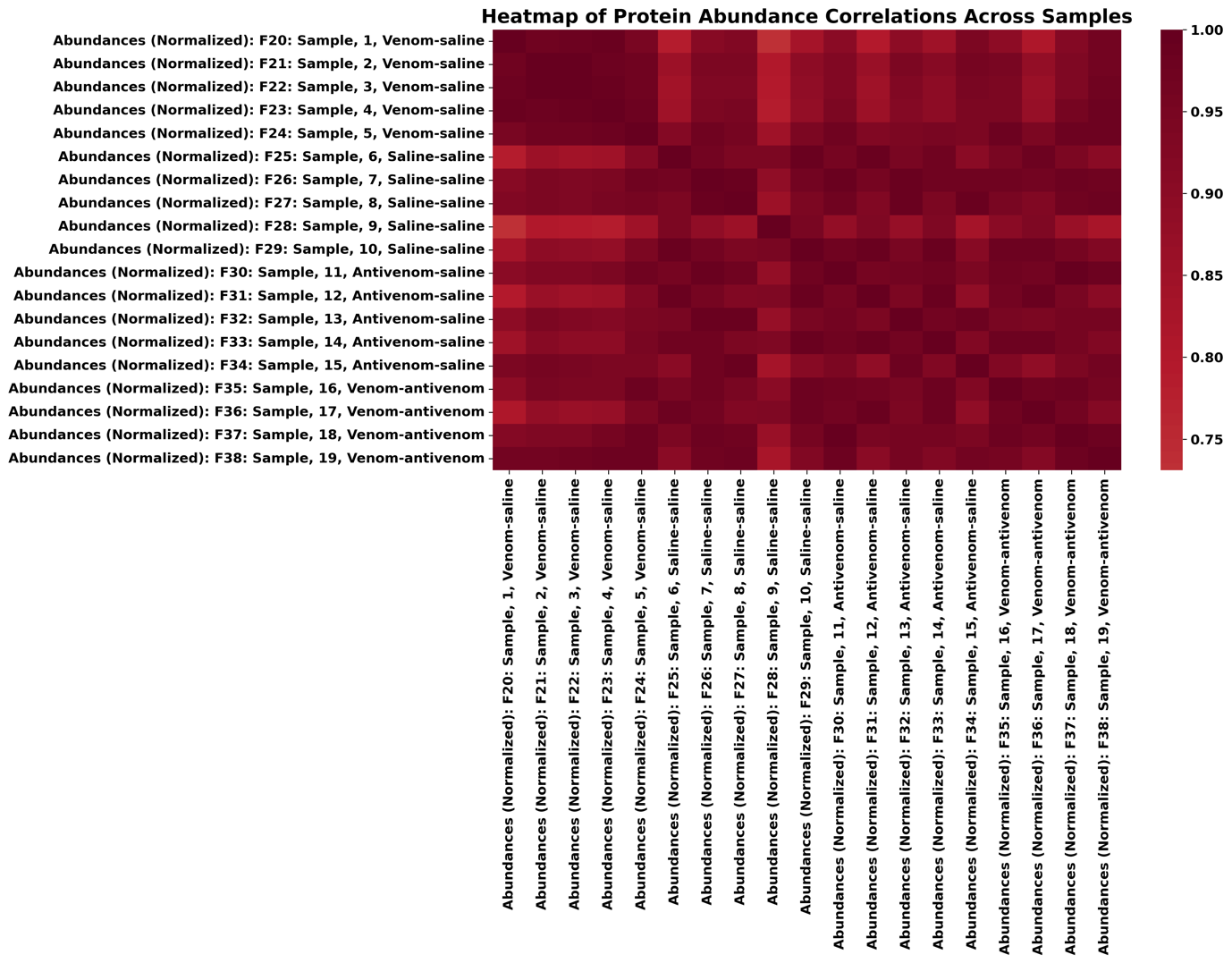


**S3. Heatmap depicting Pearson correlation coefficients for protein abundance profiles across 19 experimental samples.**

Each cell represents the correlation between pairs of samples, with the color intensity reflecting the correlation magnitude from a minimum of 0.75 to a maximum of 1.00, as indicated by the color scale. Sample labels on the y-axis and x-axis correspond to the treatment type and sample number, ranging from F20 to F38, with treatments including venom-saline, saline-saline, antivenom-saline, and venom-antivenom combinations. The prevalent red hues across the heatmap suggest a predominantly positive correlation across the sample set, hinting at consistent protein expression patterns under the conditions studied.
