# Supplementary material for: Bioinformatics-Guided Identification and Quantification of Biomarkers of Crotalus atrox Envenoming and Its Neutralization by Antivenom
Source: Mol Cell Proteomics. 2025 Mar 25;24(5):100956. doi: 10.1016/j.mcpro.2025.100956 (PMC12140956; doi:10.1016/j.mcpro.2025.100956)
Supplement: Suplimmentary File 4 [file mmc4.docx]

Table 1. Top 17 upregulated and downregulated protein markers sensitive to antivenom treatment

| No. | Accession | Protein name | Biological Process |
| --- | --- | --- | --- |
| 1 | A2AGL3↑ | Ryanodine receptor 3 | calcium ion transmembrane transport [GO:0070588], calcium ion transport [GO:0006816], cellular calcium ion homeostasis [GO:0006874], cellular response to ATP [GO:0071318}, cellular response to caffeine [GO:0071313], cellular response to calcium ion [GO:0071277], cellular response to magnesium ion [GO:0071286], protein homotetramerization [GO:0051289], striated muscle contraction [GO:0006941] |
| 2 | P04202↑ | Transforming Growth Factor Beta 1 | regulation of transforming growth factor beta receptor signaling pathway [GO:0017015], negative regulation of cell-cell adhesion [GO:0022408], cellular response to virus [GO:0098586] |
| 3 | Q9CYH2↑ | Peroxiredoxin Like 2A | regulation of osteoclast differentiation [[GO:0045670](https://www.ebi.ac.uk/QuickGO/term/GO:0045670)], cellular oxidant detoxification [[GO:0098869](https://www.ebi.ac.uk/QuickGO/term/GO:0098869)], |
| 4 | Q9WUM4↑ | Coronin-1C | activation of GTPase activity [GO:0090630], regulation of fibroblast migration [GO:0010762], endosomal transport [GO:0016197], phagocytosis [GO:000690] |
| 5 | Q9WUZ5 ↑ | troponin I1 | cardiac muscle contraction [GO:0060048], skeletal muscle contraction [GO:0003009], transition between fast and slow fiber [GO:0014883], ventricular cardiac muscle tissue morphogenesis [[GO:0055010](https://www.ebi.ac.uk/QuickGO/term/GO:0055010)], regulation of system process [ [GO:0044057](https://www.ebi.ac.uk/QuickGO/term/GO:0044057)] |
| 6 | P70694**↓** | aldo-keto reductase family 1, member C6 | steroid biosynthetic process [GO:0006694], daunorubicin metabolic process [GO:0044597], doxorubicin metabolic process [GO:0044598], progesterone metabolic process [GO:0042448], prostaglandin metabolic process [GO:0006693], lipid metabolic process [GO:000662], steroid biosynthetic process [GO:0006694], steroid metabolic process [GO:0008202], negative regulation of mRNA splicing, via spliceosome [GO:0048025] |
| 7 | B1AUY8**↓** | N-α-acetyltransferase 10 | No Biological Process Found (https://www.ebi.ac.uk/QuickGO/annotations?geneProductId=B1AUY8) |
| 8 | P48962**↓** | ADP/ATP translocase 1 | adaptive thermogenesis [GO:1990845]; ADP transport [GO:0015866]; apoptotic mitochondrial changes [GO:0008637]; mitochondrial ADP transmembrane transport [GO:0140021]; mitochondrial ATP transmembrane transport [GO:1990544]; negative regulation of cardiac muscle cell apoptotic process [GO:0010667]; |
| 9 | Q3UZZ6 **↓** | sulfotransferase family 1D, member 1 | sulfation [GO:0051923], catecholamine metabolic process [GO:0006584], lipid metabolic process [GO:0006629], sulfate assimilation [GO:0000103] |
| 10 | P62242**↓** | 40S ribosomal protein S8 | cytoplasmic translation [GO:0002181]; maturation of SSU-rRNA from tricistronic rRNA transcript (SSU-rRNA, 5.8S rRNA, LSU-rRNA) [GO:0000462] |
| 11 | Q8BH35 ↑ | Complement C8 Beta Chain | complement activation [[GO:0006956](https://www.ebi.ac.uk/QuickGO/term/GO:0006956)], immune response [[GO:0006955](https://www.ebi.ac.uk/QuickGO/term/GO:0006955)], innate immune response [GO:0045087], killing of cells of another organism [GO:0031640], immune system process [GO:0002376], complement activation, alternative pathway [GO:0006957], complement activation, classical pathway [GO:0006958], positive regulation of immune response [GO:0050778] |
| 12 | Q9EQS3 ↑ | MYC Binding Protein | regulation of DNA-templated transcription [GO:0006355],positive regulation of DNA-templated transcription [ GO:0045893], |
| 13 | P41317**↓** | mannose binding lectin 2 | positive regulation of phagocytosis [GO:0050766], surfactant homeostasis [GO:0043129], innate immune response [GO:0045087], complement activation, lectin pathway [GO:0001867], immune system process [GO:0002376], complement activation, classical pathway [GO:0006958] |
| 14 | E9PUM5↑ | Complement Factor H Related 4 | complement activation [GO:0006956], regulation of complement activation [GO:0030449], regulation of complement activation, alternative pathway [GO:0030451], proteolysis [GO:0006508] |
| 15 | D3Z5I1**↓** | Zinc Finger CCCH-Type Containing, Antiviral 1 | negative regulation of viral genome replication [GO:0045071], positive regulation of mRNA catabolic process [GO:006101], response to virus [GO:0009615] |
| 16 | Q8BH35↑ | Complement component C8 beta chain | complement activation [GO:0006956]; complement activation, alternative pathway [GO:0006957]; complement activation, classical pathway [GO:0006958]; cytolysis [GO:0019835]; positive regulation of immune response [GO:0050778] |
| 17 | Q8BMS1**↓** | Trifunctional enzyme subunit alpha | cardiolipin acyl-chain remodeling [GO:0035965]; fatty acid beta-oxidation [GO:0006635]; response to insulin [GO:0032868]; response to xenobiotic stimulus [GO:0009410] |
