# Supplementary material for: Bioinformatics-Guided Identification and Quantification of Biomarkers of Crotalus atrox Envenoming and Its Neutralization by Antivenom
Source: Mol Cell Proteomics. 2025 Mar 25;24(5):100956. doi: 10.1016/j.mcpro.2025.100956 (PMC12140956; doi:10.1016/j.mcpro.2025.100956)
Supplement: Suplimmentary File 5 [file mmc5.docx]

Supplementary materials S5: Volcano plots showing similar significant proteins sensitive to the administration of antivenom, venom, or antivenom + venom complexes


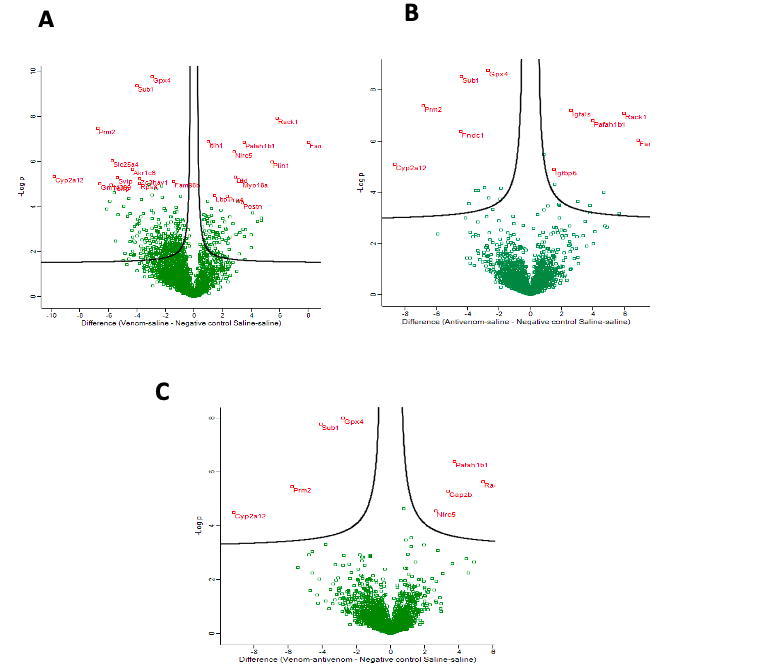


**Figure S2:** Volcano plots showing similar significant proteins sensitive to the administration of antivenom, venom, or antivenom + venom complexes, depicting a possible immune response to the foreign proteins (from antivenom IgG, venom toxins, or IgG + toxin complex) which is the beginning of hypersensitivity reactions, especially to foreign IgG. (A) Significant proteins to the antivenom-alone group compared with the negative control group. (B) Significant proteins sensitive to both venom + antivenom groups. (C) Significant proteins sensitive venom-alone group compared with negative control
